# Migraine: Epidemiology, Risk Factors, Pathophysiology, and Treatment

**DOI:** 10.1002/mco2.70933

**Published:** 2026-08-30

**Authors:** Weiwei Lu, Yuan Zhang, Chen Shi, Yu Tao, Shaoxin Li, Yufang Sun, Fuhai Ji, Dongsheng Jiang, Terrance P. Snutch, Jin Tao

**Affiliations:** ^1^ The Fourth Affiliated Hospital of Soochow University, Suzhou Dushu Lake Hospital, Suzhou Medical College of Soochow University, Soochow University Suzhou China; ^2^ The First Affiliated Hospital of Soochow University, School of Basic Medical Sciences, Suzhou Medical College of Soochow University Suzhou China; ^3^ Jiangsu Key Laboratory of Drug Discovery and Translational Research For Brain Diseases Centre For Ion Channelopathy Soochow University Suzhou China; ^4^ The Third Affiliated Hospital of Soochow University, The First People's Hospital of Changzhou Changzhou China; ^5^ Clinical Research Center of Neurological Disease Department of Geriatrics The Second Affiliated Hospital of Soochow University Suzhou China; ^6^ Department of Anesthesiology The First Affiliated Hospital of Soochow University Suzhou China; ^7^ Precision Research Center For Refractory Diseases Shanghai General Hospital, Shanghai Jiao Tong University School of Medicine Shanghai China; ^8^ Michael Smith Laboratories University of British Columbia Vancouver British Columbia Canada; ^9^ Biomedical Basic Research Center of Jiangsu Soochow University Suzhou China

**Keywords:** CGRP, epidemiology, glial heterogeneity, migraine, neuroinflammation, precision medicine

## Abstract

Migraine is a common, disabling brain disorder that affects about 1 billion people worldwide and disproportionately burdens women and working‐age adults. Once viewed mainly as a vascular headache, migraine is now understood as a heterogeneous neurobiological syndrome arising from interactions among genetic susceptibility, hormonal influences, environmental exposures, and cortical, brainstem, trigeminovascular, and neuroinflammatory networks. Although calcitonin gene‐related peptide (CGRP)‐pathway therapies, gepants, and neuromodulation have transformed care, major gaps remain in disease stratification, prevention of chronification and management of special populations. Here, we review recent evidence on global and regional epidemiology, sex‐specific and environmental risk factors, and the transition from episodic to chronic migraine. We then examine current mechanistic models, focusing on cortical spreading depolarization, trigeminovascular signaling, CGRP biology, neurogenic inflammation, glial activation, and brain network dysfunction. We also assess acute and preventive treatment strategies, including ditans, gepants, monoclonal antibodies, and onabotulinumtoxinA, and discuss persistent challenges in pediatric, geriatric, and pregnancy‐associated migraine care. By integrating population, mechanistic and therapeutic perspectives, this review reframes migraine as a stratified brain network disorder rather than a uniform vascular pain syndrome. This synthesis highlights priorities for biomarker development, precision medicine, and more equitable implementation of effective migraine care.

## Graphical abstract

Migraine is a highly prevalent, female‐predominant, and disabling brain disorder shaped by genetic susceptibility, hormonal influences, modifiable attack factors, and comorbidity‐related progression. This review integrates current evidence on migraine epidemiology, phase‐specific neurovascular and neuroglial mechanisms, including CGRP signaling and neuroglial amplification, and the transition from episodic to chronic disease. It further highlights how treatment is evolving from empirical symptom relief toward mechanism‐based acute, preventive, nonpharmacological, and emerging precision‐oriented strategies.

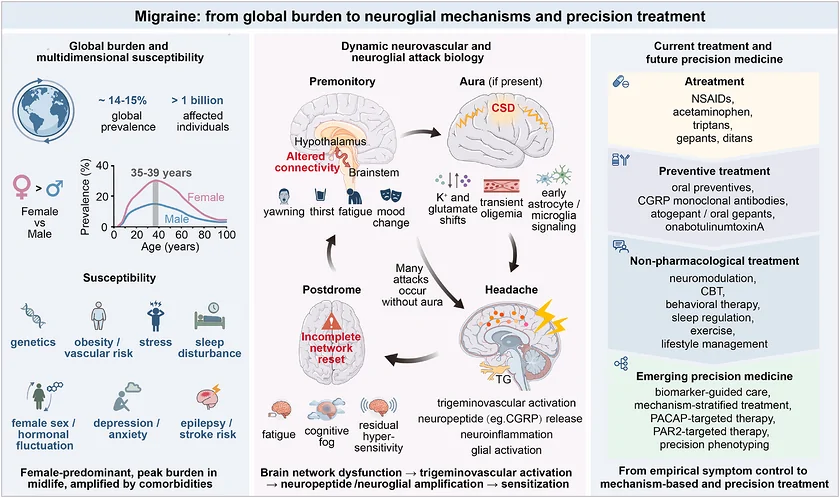

## 1 Introduction

Migraine is a complex, recurrent primary headache disorder characterized by moderate‐to‐severe, often pulsating head pain that is commonly accompanied by photophobia, phonophobia, nausea, and vomiting [1, 2]. It is a distinct neurological disease driven by multifactorial pathophysiological mechanisms rather than a secondary symptom [3]. Contemporary concepts have moved beyond a purely vascular model and now frame migraine as a disorder involving interacting cortical, brainstem, trigeminovascular, and neuroinflammatory systems [1, 4]. Globally, migraine affects roughly one billion people, with current best estimates of prevalence around 14–15%, and it imposes substantial personal, societal, and economic burdens [1, 5, 6].  Its prevalence is nearly three times higher in women than in men [5], and it peaks during the most productive decades of life, particularly between 20 and 50 years of age [7].

Defining migraine and its subtypes is essential for accurate diagnosis, interpretation of clinical heterogeneity, and appropriate treatment selection. According to the International Classification of Headache Disorders, third edition, migraine is categorized into several subtypes, most notably migraine without aura (MwoA) and migraine with aura (MwA), each with distinct diagnostic criteria [8, 9]. Chronic migraine (CM), defined as headache occurring on ≥15 days per month for >3 months, with ≥8 days meeting migraine criteria, further complicates the clinical picture [1, 7]. Approximately 2.5% of individuals with episodic migraine progress to CM each year [7]. This classification framework standardizes diagnosis, facilitates communication across clinical and research settings, and informs treatment selection, as different migraine subtypes can show distinct patterns of burden and therapeutic response.

The burden of migraine extends beyond the individual to families, workplaces, health systems, and societies. Migraine remains one of the leading causes of nonfatal disability worldwide and substantially diminishes quality of life across personal, social, and occupational domains [10, 11].  Global Burden of Disease (GBD) analyses continue to show a major contribution of migraine to years lived with disability (YLDs) [5, 6, 12], with particularly high burden among women and working‐age adults [12]. Despite this burden, clinical management remains suboptimal. Recent multicountry survey data show that most individuals who meet criteria for preventive treatment are not using it [13], and fewer than 15% of people with migraine who need care successfully traverse the combined barriers of consultation, diagnosis, and minimally appropriate pharmacologic treatment [14]. These findings underscore the urgent need for more effective, accessible, and equitable migraine care, particularly for adolescents and women of reproductive age, who bear a disproportionate share of disease burden.

This review prioritizes literature published in the last 3 years, while incorporating foundational studies where necessary, and addresses multiple dimensions of migraine. It covers global and regional epidemiology; genetic, environmental, and sex‐specific risk factors; and complex pathophysiological mechanisms, including spreading depolarization and neurogenic inflammation [15]. We also evaluate recent evidence for acute and preventive treatment options, including the dual use of gepants and the efficacy of monoclonal antibodies, while addressing management challenges in pediatric, geriatric, and pregnant populations [15, 16]. To ensure comprehensive and systematic coverage of the recent literature, we used a structured search strategy. PubMed was searched for relevant studies published up to June 2026, with emphasis on literature from 2023 onward. Search terms included “migraine,” “epidemiology,” “risk factors,” “pathophysiology,” “neuroinflammation,” “glia,” and “therapy.” Screening involved title and abstract review followed by full‐text assessment against predefined inclusion criteria: original research articles, meta‐analyses, clinical guidelines, consensus statements, and authoritative reviews relevant to migraine epidemiology, mechanisms, comorbidities, imaging, and treatment. This strategy was designed to capture high‐quality evidence across clinical, mechanistic, therapeutic, and imaging domains, including consensus‐based imaging guidance relevant to differentiating migraine from vascular mimics [17].

## 2 Epidemiology and The GBD

### 2.1 Global and Regional Prevalence

Migraine is among the most prevalent neurological disorders worldwide and remains a leading cause of nonfatal health loss [5]. Current estimates place the global age‐standardized prevalence of migraine at approximately 14–15%, corresponding to more than 1 billion affected individuals [5, 15, 18]. From a public‐health perspective, however, prevalence alone does not fully capture disease impact; migraine is also a major contributor to disability burden and is the leading cause of YLDs among women of reproductive age [15]. Although prevalence varies across regions and population subgroups, cross‐study comparisons require caution because estimates are influenced by case definition, inclusion of probable migraine, sampling strategy, and survey methodology [15].

Recent analyses based on GBD 2021 and subsequent updates indicate that the absolute number of people living with migraine has increased substantially since 1990, reaching approximately 1.16 billion cases in 2021 [12, 19]. Importantly, this rise in case counts should not be interpreted as a proportional increase in age‐standardized prevalence alone. Rather, much of the increase reflects population growth, population aging, and improved epidemiological ascertainment, including broader and more standardized case identification [5, 19]. Consistent with this interpretation, the most recent GBD 2023 headache analysis shows that the global age‐standardized prevalence of migraine has remained broadly stable, at 14.1% (95% uncertainty interval 12.1–15.9), over the past 3 decades [12]. Thus, the contemporary epidemiological message is one of persistently high global burden rather than unequivocally rising age‐standardized prevalence.

**FIGURE 1:**
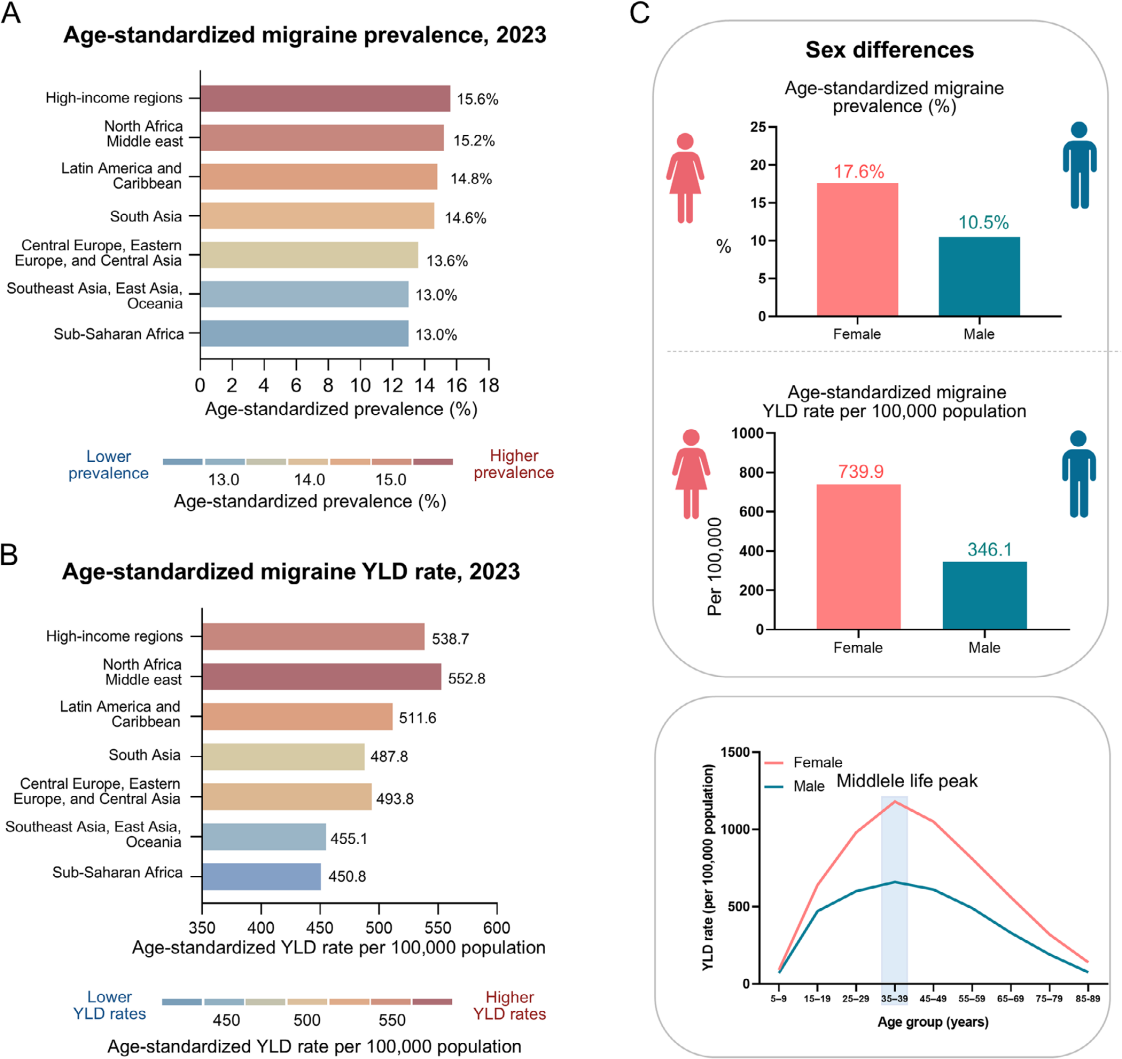
Global prevalence and disability burden of migraine: regional distribution and sex differences. This figure summarizes the global epidemiology of migraine using data from the Global Burden of Disease (GBD) 2023 as discussed in this review. (A) Age‐standardized migraine prevalence across major world regions. (B) Age‐standardized migraine YLD rate across major world regions. (C) Sex differences in migraine burden, highlighting the substantially greater burden in females than in males; the panel may also illustrate the midlife peak in burden, if applicable. Overall, migraine remains highly prevalent worldwide, whereas regional variation is more pronounced for disability burden than for prevalence alone. *Abbreviation*: YLDs, years lived with disability.

Marked regional heterogeneity is evident, but regional contrasts should be interpreted cautiously because data availability and methodological consistency remain uneven. Earlier GBD‐based analyses suggested substantial geographical variation, with high prevalence estimates in some parts of South and Southeast Asia and lower estimates in China [19]. More recent GBD 2023 super‐region analyses indicate that age‐standardized migraine prevalence is broadly similar across major world regions, with overlapping uncertainty intervals, whereas the burden measured by YLDs shows clearer regional differentiation [12]. In 2023, the highest age‐standardized YLD rate for migraine was observed in North Africa and the Middle East, followed closely by high‐income regions, whereas the lowest rate was reported in sub‐Saharan Africa [12]. These patterns suggest that differences in symptomatic time, disability, and access to care may contribute to burden beyond prevalence alone. Accordingly, apparently lower migraine prevalence in some low‐ and middle‐income settings may partly reflect underdiagnosis, limited access to health care, and incomplete epidemiological capture rather than a genuinely lower disease burden [15, 20] (Figure 1).

Population‐level variation by sex and age is one of the most robust findings in migraine epidemiology. Across regions, migraine is approximately twofold to threefold more common in females than in males, with recent global estimates showing an age‐standardized prevalence of 17.6% in females versus 10.5% in males [12, 21] (Figure 1). This sex disparity is reflected not only in prevalence but also in disability burden: headache‐attributable YLD rates are more than twice as high in females as in males [12]. Migraine prevalence rises from childhood through adolescence, peaks in midlife, typically around 35–39 years and then declines in older age [15, 22]. Age‐period‐cohort analyses further suggest that temporal trends differ across sociodemographic settings, although such findings should be interpreted cautiously because they remain sensitive to model assumptions and ascertainment methods [23]. Children and adolescents also contribute substantially to the global burden, but pediatric estimates are especially vulnerable to diagnostic under‐recognition because probable migraine and undifferentiated headache are inconsistently captured in epidemiological surveys [15, 24].

### 2.2 Incidence, Natural History, and Disease Progression

Migraine incidence rates exhibit distinct variations, peaking in adolescence and early adulthood, particularly among females. In males, incidence peaks between 10 and 14 years, while prevalence and disability‐adjusted life years (DALYs) peak in the 30–44 years age group [25]. Among adolescents and young adults, incidence and prevalence have increased markedly, with females consistently showing higher rates [26].

Global projections indicate continued growth in migraine prevalence through 2050. Disease progression is a critical clinical metric; annually, approximately 2.5% of patients with episodic migraine progress to CM [7, 27]. This transition is heavily influenced by psychiatric comorbidities. Individuals with CM are approximately twice as likely as those with episodic migraine to suffer from depression (30%) or anxiety (30%) [7, 28]. Furthermore, the burden of progression is exacerbated by migraine‐related stigma, which correlates with increased disability, less care‐seeking, and poorer treatment outcomes [15, 29]. Genetic factors also contribute to progression; individuals with a family history often present with earlier onset, suggesting genetic anticipation [30]. Finally, hormonal fluctuations contribute to progression patterns, as prevalence typically peaks during reproductive years and declines postmenopause [12, 31].

### 2.3 Multidimensional Burden Assessment

The burden of migraine is multidimensional, encompassing health‐related quality of life impairment, disability, socioeconomic costs, and comorbidity‐related exacerbation, with impacts felt at the individual, family, and societal levels [1]. DALYs and YLD are critical metrics for quantifying this impact; globally, migraine is the second leading cause of YLDs across all age groups and the premier cause of disability in women aged 16–49 years [1, 3, 15]. This burden is heavily concentrated among adolescents and young adults [32]. In Asia, the highest age‐standardized DALYs rates for migraine are observed in Southeast Asia [33], while in Europe, the burden remains high due to persistent prevalence among women and middle‐aged adults [34].

Comorbidities significantly amplify the multidimensional burden of migraine, as the disorder rarely occurs in isolation. Common comorbidities include psychiatric disorders (anxiety, depression), pain syndromes (neck pain, temporomandibular disorders [TMD]), sleep disturbances, and fibromyalgia [35]. These comorbidities not only exacerbate migraine symptoms but also contribute independently to disability and diminished QoL. Notably, patients with CM are approximately twice as likely as those with episodic migraine to suffer from depression (30 vs. 17%) or anxiety (30 vs. 19%) [7, 15]. Furthermore, migraine‐related stigma represents a significant interictal burden, correlating with increased disability, decreased quality of life, and reduced care‐seeking behavior [7, 36]. Medical comorbidities such as hypertension (34%), hyperlipidemia (34%), and obesity (26%) are also significantly more prevalent in CM populations [7].

The socioeconomic burden of migraine is substantial, driven by reduced work productivity, absenteeism, and healthcare utilization. Disability and work productivity loss are particularly significant in young and middle‐aged adults, the most economically active segment of the population [37]. The cumulative effect of migraine and its comorbidities leads to marked reductions in multiple domains of QoL, including physical functioning, emotional well‐being, social interactions, and occupational productivity. The Migraine‐Specific Quality of Life Questionnaire scores are notably reduced in patients with coexisting depression or anxiety, reflecting the profound impact on daily activities [38]. Sleep disorders further compound this burden by triggering migraine attacks and impairing cognitive function, creating a vicious cycle [39, 40].

Migraine also exhibits a significant epidemiological link with cerebrovascular diseases, adding another dimension to its burden. Migraine, particularly MwA, is associated with an increased risk of ischemic cerebrovascular events, transient ischemic attacks (TIAs), and cervical artery dissection [41, 42]. A large systematic review and meta‐analysis found that migraine markedly increases the odds of all strokes and TIAs during pregnancy and the puerperium period [41], while migraineurs with aura are more prone to vascular risk factors such as hypertension and hyperlipidemia [43]. Neuroimaging frequently reveals incidental T2‐hyperintense deep white matter lesions in migraineurs, indicative of vascular risk [17]. However, it is clinically vital to distinguish these from inflammatory mimics; while white matter lesions are common in migraine and aging, cortical lesions—frequently observed in multiple sclerosis—have not been observed in migraine patients [17].

### 2.4 Epidemiology in Specialized Populations

#### 2.4.1 Pediatrics and Adolescence

Migraine in pediatric and adolescent populations (aged 5–24 years) exhibits distinct epidemiological and clinical characteristics compared with adults, presenting unique challenges in diagnosis and management. Migraine is common in children, and its prevalence increases significantly during the transition through adolescence [44, 45]. Globally, migraine cases among youths and young adults aged 15–39 years increased by nearly 40% from 1990 to 2021, reaching 593.8 million cases in 2021 [46, 47]. Among children and adolescents aged 5–19 years, prevalence rose by over 20% from 1990 to 2021, with the highest prevalence observed in the 15–19 years age group [12, 48]. Incidence is particularly high in adolescents: among adolescents and young adults (AYA, aged 10–24 years), incidence and prevalence have increased markedly, with females consistently showing higher rates than males [12, 48]. Notably, migraine is identified as the leading cause of disability in individuals younger than 50 years, emphasizing the high burden on these younger cohorts.

Clinically, pediatric migraine often presents with atypical features that complicate diagnosis. While adult attacks typically last 4–72 h [4], the duration of migraine attacks in children can be shorter, ranging from 2 to 72 h [16]. Children under 7 years frequently exhibit bilateral pain rather than the unilateral pattern seen in adults, and the headache is often nonpulsating [16]. Gastrointestinal disturbances, such as abdominal pain and vomiting, are commonly prominent [16]. Diagnosis is further hindered by difficulties in symptom description among younger children; consequently, reliable accounts from parents regarding clinical features and lifestyle factors are essential for accurate assessment [16]. Gender differences emerge postpuberty, with prevalence increasing in girls due to hormonal influences [11]. Pediatric migraine also shows a strong association with family history, reflecting a heritability of approximately 40% [4]. Undiagnosis and undertreatment remain significant issues, highlighting the need for age‐appropriate diagnostic criteria and tailored management strategies.

#### 2.4.2 Older Adults

The epidemiology of migraine in the geriatric population (typically defined as adults aged 65 years and older) is characterized by declining prevalence but persistent disability. While many epidemiological studies are restricted to individuals aged 18–65 years, leaving data for those over 60 years relatively sparse [44], current evidence indicates that prevalence peaks between 35 and 39 years [44]. Prevalence subsequently declines to approximately 7% in individuals aged 60–69 years and to approximately 4% in those aged 70 years or older [44]. The clinical presentation often differs from younger populations: older adults may experience fewer attacks, but these may present with less typical features—such as aura without headache—and higher rates of comorbidities, including hypertension and cardiovascular disease [5, 44].

Age‐related neurobiological changes, hormonal fluctuations, and alterations in vascular and neuronal function contribute to the decline in migraine frequency and severity, though the mechanisms of this remission remain under investigation [44]. However, comorbidities and polypharmacy complicate management; older adults often require individualized treatment due to the risk of adverse drug reactions and drug–drug interactions [16, 44]. For example, the use of nonsteroidal anti‐inflammatory drugs (NSAIDs) may be limited by gastrointestinal or cardiovascular risks, while pharmacokinetic studies of preventive therapies (e.g., galcanezumab) indicate minimal age‐related differences in efficacy and safety up to 65 years [45]. Cognitive impairment and atypical headache presentations can also lead to misdiagnosis or delayed diagnosis, further impacting outcomes [49].

#### 2.4.3 Sex and Gender Differences

Sex differences in migraine epidemiology are well established. Migraine is three times more common in females than in males [4, 50]. This disparity emerges around puberty and persists throughout reproductive years, primarily attributed to hormonal influences [11]. The global age‐standardized prevalence of migraine is significantly higher among females (17.6%) than males (10.5%) [12]. Furthermore, YLD rates are more than twice as high in females as in males [2]. Estrogen fluctuations are a key driver: estrogen withdrawal triggers migraine attacks (particularly menstrual migraine), while stable estrogen levels during pregnancy often improve symptoms [50, 51]. Women also experience more severe migraine symptoms, including higher attack frequency, longer duration, and greater disability, compared with men [52].

Beyond biological sex, gender‐related factors—including identity and social disparities—influence migraine epidemiology in sexual and gender minorities (SGM). While limited data focus specifically on SGM populations, gender‐based social stressors, such as stigma and discrimination, may act as triggers and exacerbate symptom severity [5]. Additionally, hormonal therapies used in gender‐affirming care may modulate susceptibility, though further research is required to elucidate these multifaceted dynamics [2]. Epidemiological data consistently confirm higher prevalence and burden in women, influenced by socioeconomic and regional factors [21, 53]. For example, in collegiate athletes, females report higher rates and more severe symptoms [54]; among US veterans, women have a threefold higher lifetime prevalence [55]. These differences have significant clinical implications, as women are more likely to seek care but face unique challenges, including the management of menstrual migraine and considerations related to hormone‐containing therapies [50, 56].

## 3 Risk Factors and Comorbidities

A clear framework is essential because migraine literature often conflates three distinct domains: baseline susceptibility determinants, modifiable attack‐related factors, and risk factors for progression from episodic to CM [11, 15, 44, 57]. Distinguishing them improves epidemiological interpretation and prevents confusion between short‐term attack precipitants and factors that influence long‐term disease course [15, 57].

Baseline susceptibility determinants are relatively stable characteristics that shape lifetime migraine risk. These include genetic architecture, biological sex, hormonal milieu, and age [11, 15]. Migraine is substantially heritable and ranges from rare monogenic syndromes to common polygenic forms, with susceptibility involving neuronal excitability, glutamatergic signaling, ion homeostasis, and vascular regulation [11]. Prevalence is higher in females, diverges after puberty, peaks in midlife, and declines with older age, although CM may persist in some individuals [15, 44].

A second category includes modifiable factors associated with attack frequency or disease expression. These are often labeled “triggers,” but this term should be used cautiously [15, 57]. Sleep disruption, stress, missed meals, alcohol, sensory overload, and hormonal fluctuation may alter attack threshold in susceptible individuals, yet they are not necessarily validated epidemiological risk factors for disease onset or progression [11, 15]. Some perceived triggers may also reflect prodromal symptoms rather than true external causes [13]. Thus, these exposures are better regarded as attack modifiers.

In contrast, chronification risk factors are variables associated with progression from episodic to CM [57]. CM is defined as headache on at least 15 days per month for more than 3 months, with at least 8 days meeting migraine criteria or responding to migraine‐specific therapy [57]. Approximately 2.5–3.0% of individuals with episodic migraine progress annually [57]. The most consistent predictors are high baseline headache frequency, medication overuse, depression, and poor acute‐treatment response [15, 57]. These factors are clinically important because they relate to disease persistence and escalation rather than the occurrence of individual attacks.

This framework also clarifies the role of comorbidities. Conditions such as anxiety, depression, obesity, sleep disorders, asthma, chronic pain syndromes, and metabolic disease may act as shared traits, burden amplifiers, or contributors to chronification risk [15, 57]. However, association should not be equated with causation, as some comorbidities may reflect shared genetic or biological mechanisms rather than direct causal effects [11, 15]. Overall, migraine risk should be interpreted at three levels: predisposing determinants establish susceptibility, attack modifiers influence short‐term attack expression, and chronification factors shape long‐term worsening (Table 1).

**TABLE 1:** Multidimensional determinants of migraine: genetic susceptibility, demographic and hormonal influences, modifiable attack factors, and chronification‐related comorbidities.

Risk factor category	Specific factors	Strength of evidence	Remarks	References
Genetic	Familial hemiplegic migraine genes: *CACNA1A, ATP1A2, SCN1A*	High	Susceptibility determinant: rare high‐effect variants disrupt ion homeostasis and glutamatergic signaling, lowering the threshold for cortical hyperexcitability and aura‐prone migraine.	[11, 58, 59]
Genetic	Common polygenic susceptibility loci/polygenic burden	High	Susceptibility determinant: cumulative common variants shape neuronal, vascular, and neuroimmune vulnerability across common migraine phenotypes.	[4, 11, 60, 61]
Genetic	Positive family history/familial aggregation	High	Susceptibility determinant: strong familial aggregation supports inherited liability and may be associated with earlier onset or stronger phenotypic expression.	[11, 58, 59]
Demographic/hormonal	Female sex	High	Susceptibility determinant: female predominance emerges after puberty and is associated with substantially greater prevalence and disability burden.	[12, 21, 50, 62, 63]
Demographic/hormonal	Puberty and reproductive years	High	Susceptibility determinant: hormonal maturation marks the divergence of female prevalence and amplifies burden during the reproductive life stage.	[11, 15, 44, 50, 62]
Demographic/hormonal	Estrogen withdrawal/menstrual cycling	High	Attack modifier: perimenstrual estrogen decline lowers attack threshold and helps explain menstrual migraine in susceptible women.	[50, 63, 64]
Demographic/hormonal	Pregnancy, perimenopause, menopause, menopausal hormone therapy	Medium	Attack modifier: relatively stable pregnancy hormones often improve attacks, whereas perimenopause and fluctuating hormone therapy may worsen them; effects are context dependent.	[63, 65, 66, 67]
Demographic/hormonal	Age/life‐course effects	High	Susceptibility determinant: prevalence rises from adolescence, peaks in midlife, and declines with older age, while phenotype and persistence vary across the lifespan.	[12, 15, 22, 44]
Lifestyle factors	Insomnia, sleep disturbance, irregular sleep	High	Attack modifier: disturbed or irregular sleep lowers the attack threshold and may bidirectionally worsen headache burden.	[39, 68, 69, 70]
Lifestyle factors	Psychological stress	Medium	Attack modifier: stress alters central stress‐homeostatic circuits and commonly increases short‐term attack susceptibility and overall burden.	[71, 72, 73, 74]
Lifestyle factors	Missed meals/fasting	Medium	Attack modifier: fasting or meal irregularity may precipitate attacks in susceptible individuals rather than acting as a uniform cause of disease onset.	[15, 75, 76]
Lifestyle factors	Alcohol, specific foods, sensory overload	Low–medium	Attack modifier: these exposures are heterogeneous and individualized, and may sometimes reflect misattributed prodromal phenomena rather than true external causes.	[15, 75, 76]
Lifestyle factors	Physical inactivity/sedentary behavior	Medium	Attack modifier: lower activity is associated with greater headache burden, whereas regular moderate exercise may be protective within comprehensive management.	[11, 68, 69, 77]
Lifestyle factors	Socioeconomic disadvantage and limited access to care	Medium	Burden amplifier/shared biology: delayed diagnosis, undertreatment, and restricted access to prevention magnify disability and may indirectly favor progression.	[13, 14, 15]
Comorbidity/progression	High baseline headache frequency	High	Chronification factor: one of the strongest predictors of transition from episodic to chronic migraine, especially when monthly headache days are already high.	[15, 57]
Comorbidity/progression	Medication overuse	High	Chronification factor: frequent acute medication use is a major modifiable predictor of worsening headache frequency and chronic migraine.	[57]
Comorbidity/progression	Depression	High	Chronification factor: reproducibly associated with progression and greater disability, likely reflecting bidirectional biopsychosocial vulnerability.	[15, 57, 71, 78, 79, 80, 81]
Comorbidity/progression	Anxiety	Medium	Burden amplifier/shared biology: common comorbidity that increases disability and may contribute to persistence, although causal certainty is less consistent than for depression.	[15, 57, 71, 78, 79, 80]
Comorbidity/progression	Obesity/metabolic disease	Medium	Burden amplifier/shared biology: linked to higher headache frequency and chronic migraine, with overlap in metabolic and vascular vulnerability.	[11, 57, 82]
Comorbidity/progression	Sleep disorders as clinical comorbidity	Medium–high	Burden amplifier/shared biology: sleep comorbidity amplifies attack burden, impairs recovery, and may contribute to progression in predisposed patients.	[39, 57, 82]
Comorbidity/progression	Chronic pain syndromes, neck pain, TMD, fibromyalgia	Medium	Burden amplifier/shared biology: overlapping pain vulnerability and sensitization worsen disability, symptom complexity, and overall interictal burden.	[15, 35, 83, 84, 85]
Comorbidity/progression	Hypertension/hyperlipidemia/vascular risk clustering	Medium	Burden amplifier/shared biology: vascular comorbidity clusters with migraine, especially aura, and adds to long‐term burden and risk stratification.	[7, 15, 43, 57, 86, 87]

*Note*: High = supported by replicated genetic studies, GBD/meta‐analytic evidence, or consistent longitudinal studies; Medium = consistent observational or Mendelian‐randomization evidence, but with limited causal certainty; Low = preliminary, heterogeneous, or mainly trigger‐based literature. Several listed comorbidities are best interpreted as shared biological traits or burden amplifiers, not proven direct causes of migraine onset.

*Abbreviation*: TMD, temporomandibular disorders.

### 3.1 Nonmodifiable Determinants

#### 3.1.1 Genetic Architecture

Genetic factors are major nonmodifiable determinants of migraine susceptibility. Current evidence supports a spectrum ranging from rare monogenic forms to common polygenic migraine, with both contributing to disease risk, phenotypic heterogeneity, and possibly treatment response. Familial aggregation is strong: in the Copenhagen Migraine Cohort, 73.4% of patients reported a positive family history of migraine, supporting a substantial heritable component [58]. Accordingly, migraine genetics is best conceptualized as comprising rare, high‐effect variants that underlie familial syndromes and numerous common, small‐effect variants that contribute to the more prevalent sporadic forms [11].

Rare monogenic variants are best established in familial hemiplegic migraine (FHM), a subtype characterized by aura with motor weakness. Pathogenic variants in CACNA1A, ATP1A2, and SCN1A are the canonical causes of FHM [88, 89, 90, 91]. These genes encode proteins that regulate neuronal or astrocytic ion homeostasis and glutamatergic neurotransmission; their disruption facilitates cortical spreading depolarization (CSD), the electrophysiological substrate of aura, and increases susceptibility to neuronal hyperexcitability [11, 88, 89, 90, 91]. Beyond FHM, additional genes have been proposed in familial MwA, including MTDH and PGCP, but their causal roles remain less certain and are not yet established to the same level as the classic FHM genes [90].

By contrast, common migraine, including MwA and MwoA, has a predominantly polygenic architecture. Genome‐wide association studies (GWAS) have identified more than 100 susceptibility loci, with the largest studies reporting at least 123 robust risk loci [59, 60]. These loci are enriched in pathways related to neuronal excitability, synaptic and glutamatergic signaling, vascular development and tone, and ion or solute homeostasis, supporting a neurovascular model of migraine pathophysiology [92]. MwA and MwoA share much of this genetic background, although subtype‐specific signals have also been reported; compared with MwoA, the genetic basis of MwA remains less completely resolved, likely because of greater heterogeneity and smaller well‐powered datasets [61, 90].

Population‐specific studies further indicate that migraine risk alleles are not fully uniform across ancestries. For example, studies in Han Chinese cohorts have identified variants associated with migraine and family history, supporting ancestry‐specific components of genetic risks [93]. Mitochondrial dysfunction has also been proposed as a contributor to migraine biology, but evidence for a primary causal role of mitochondrial DNA variants in common migraine remains suggestive rather than definitive [94]. In parallel, migraine shows meaningful genetic overlap with several neurologic disorders, particularly epilepsy, consistent with shared channelopathy‐related mechanisms involving genes such as SCN1A and KCNMA1 [95]. Importantly, migraine also shares significant genetic liability with major depressive disorder, contrary to the view that overlap is minimal [96]. Overall, the genetic architecture of migraine is heterogeneous and multilayered, spanning rare pathogenic variants and a broad polygenic burden that together shape individual susceptibility [11, 61, 97].

#### 3.1.2 Biological Sex and Hormonal Influences

Biological sex and hormonal milieu are key nonmodifiable determinants of migraine and help explain its marked female predominance. Migraine is approximately two to three times more common in women than in men, with the sex gap emerging around puberty and persisting through the reproductive years [50, 62]. This pattern strongly implicates sex hormones, especially estrogen, in migraine susceptibility. Current evidence indicates that hormonal fluctuations modulate trigeminovascular signaling and calcitonin gene‐related peptide (‌CGRP)‐related pathways, although these mechanisms are complex and not exclusively estrogen dependent [63, 98].

Estrogen withdrawal is the best supported hormonal mechanism for menstrual migraine. Perimenstrual attacks occur in temporal association with the late luteal decline in estrogen, and this fall is thought to lower the threshold for migraine in susceptible individuals [64]. Menstruation‐associated prostaglandin production may also contribute to attack generation, but its mechanistic role should be described more cautiously than a direct causal pathway [70]. Across the life course, migraine typically worsens after menarche, often improves during pregnancy when estrogen levels are relatively stable and high, and may worsen during perimenopause when hormonal fluctuations become more erratic [63, 65]. After natural menopause, migraine—especially MwoA—often improves, although this pattern is not universal.

The effects of menopausal hormone therapy are variable and should be individualized. Some women improve when stable estrogen levels are achieved, but others worsen, particularly with fluctuating or oral regimens [66]. For symptomatic menopausal women with migraine, transdermal estrogen combined with progesterone is generally preferred because it provides more stable hormone levels; however, vascular risk assessment remains essential, especially in women with MwA or other stroke risk factors [66, 67]. Thus, hormone therapy should not be presented as uniformly reducing migraine frequency, but rather as having context‐dependent effects that require individualized clinical evaluation.

Other sex‐related mechanisms may also influence migraine, although the evidence is less definitive. Prolactin has emerged as a potentially female‐selective pronociceptive mediator in experimental and translational studies, but current human data are insufficient to regard it as an established clinical determinant of migraine severity [99, 100]. Likewise, sex hormones may influence mitochondrial function and nociceptor signaling [31], and hormone‐sensitive ion channels such as TRPM3 are of mechanistic interest [101]. However, these pathways should be framed as emerging rather than definitive explanations for female predominance in migraine. Overall, the strongest conclusion is that sex‐specific hormonal dynamics—particularly fluctuations in estrogen—substantially shape migraine susceptibility and clinical expression across the female life course.

#### 3.1.3 Age and Life‐Course Effects

Age is a major nonmodifiable determinant of migraine susceptibility, clinical expression, and long‐term disease course. Epidemiological data consistently show that migraine is strongly age‐related: incidence is highest in early adult life, most individuals report disease onset before 35 years of age, and prevalence rises from adolescence to a peak at approximately 35–39 years before declining thereafter [15, 44]. In population‐based studies, the overall incidence of migraine decreases with advancing age, and new‐onset migraine becomes distinctly less common after midlife [44]. This age pattern indicates that migraine is not a static disorder, but an evolving condition whose burden and phenotype vary across the life course.

The clinical phenotype of migraine also changes with age. In childhood and adolescence, prevalence is lower than in midlife, but increases steadily, with the sex gap emerging from late childhood and early adolescence [15, 44]. During adulthood, migraine reaches its greatest population burden in the most productive years of life, a feature that contributes substantially to its disability impact [44]. In later life, episodic migraine often becomes less frequent and may remit, whereas CM is more likely to persist [44]. Importantly, remission is not universal: longitudinal data suggest heterogeneous trajectories, including remission, persistence, and progression [44]. Thus, age should be considered not only a demographic correlate of migraine prevalence, but also a determinant of disease evolution.

Older age is associated with additional diagnostic and clinical complexity. Among individuals older than 60 years, migraine prevalence is substantially lower than in midlife, but the disorder remains clinically relevant, particularly because presentation often becomes less stereotypical [15, 44]. Headache may become more often bilateral, less pulsatile, or less severe, whereas aura without headache becomes more common [44]. At the same time, the background prevalence of secondary headache disorders, vascular disease, polypharmacy, and medical comorbidity increases, complicating diagnosis and management [44]. Therefore, age influences migraine not only through prevalence and remission patterns, but also through phenotype, differential diagnosis, and therapeutic context across the lifespan.

### 3.2 Modifiable Risk Factors and Disease Progression

#### 3.2.1 Lifestyle, Behavioral, and Environmental Factors

Lifestyle and behavioral factors are important modifiable influences on migraine expression, but they should be distinguished from validated risk factors for long‐term disease progression. In clinical and epidemiological studies, psychological stress, sleep disturbance, dietary exposures, physical inactivity, and some environmental stimuli are commonly associated with attack occurrence or attack burden. However, these factors are best regarded as attack modifiers rather than uniform causal determinants of migraine onset, because their effects are heterogeneous, strongly intraindividual, and often context‐dependent [15, 98]. This distinction is important to avoid conflating patient‐reported “triggers” with established predictors of chronification.

Psychological stress is among the most frequently reported attack‐related factors and is plausibly linked to migraine through dysregulation of central stress circuits, altered hypothalamic signaling, and facilitation of trigeminovascular activation [71]. Stress‐related biological responses can also interact with inflammatory signaling, but mechanistic claims should be framed cautiously: although experimental models suggest that stress may promote release of proinflammatory mediators and glia‐related signaling relevant to migraine biology, direct translation of these findings to human migraine pathogenesis remains incomplete [74]. Therefore, stress is best described as a clinically important modifier of attack susceptibility and burden rather than as a singular mechanistic driver of migraine chronification. Consistent with this interpretation, behavioral interventions such as cognitive‐behavioral therapy (CBT) and mindfulness‐based strategies can reduce headache burden in selected patients, particularly when stress is a prominent component of the clinical phenotype [72, 73].

Sleep disturbance is another major modifiable factor. Insomnia, irregular sleep–wake schedules, and sleep deprivation are consistently associated with greater migraine frequency and severity, and the relationship appears bidirectional: disturbed sleep may lower the threshold for attacks, whereas migraine itself can impair sleep continuity [68, 69]. Recent Mendelian‐randomization analyses strengthen this association by supporting a likely causal contribution of insomnia to migraine risk [69]. Accordingly, sleep regulation should be considered a core component of migraine management. Optimization of sleep hygiene is reasonable clinical advice, although evidence is stronger for the treatment of specific sleep disorders than for generic sleep‐hygiene measures alone. In particular, recognition and treatment of comorbid sleep disorders may improve overall migraine outcomes [70].

Dietary factors are frequently reported as precipitants, but the evidence base is mixed and highly individualized. Commonly cited exposures include alcohol, fasting or skipped meals, and certain foods such as chocolate or aged cheese; however, retrospective trigger attribution is vulnerable to recall bias and may also reflect misinterpretation of prodromal symptoms as causal exposures [75]. Thus, broad statements about universal dietary triggers should be avoided. Similarly, evidence for specific nutrients remains preliminary. For example, observational data suggesting an L‐shaped association between niacin intake and migraine risk are hypothesis‐generating rather than sufficient to support specific nutritional recommendations [102]. By contrast, structured dietary approaches, including Mediterranean‐style diets or ketogenic interventions, may benefit some patients, particularly when aligned with comorbid metabolic features, but current evidence remains insufficient to endorse one dietary pattern as broadly superior for migraine prevention [68, 76, 103]. A pragmatic and evidence‐consistent approach is individualized identification of reproducible dietary precipitants rather than indiscriminate elimination diets [104, 105].

Physical activity has a similarly nuanced relationship with migraine. Regular moderate aerobic activity is generally associated with lower headache burden and improved overall health, whereas vigorous exertion can precipitate attacks in a subset of individuals [68, 77]. Genetic epidemiology also suggests that sedentary behaviors and adverse vascular traits may contribute to migraine susceptibility, although mediation pathways remain incompletely defined [11]. Exercise‐based interventions are therefore best framed as part of comprehensive lifestyle management rather than as migraine‐specific monotherapy. In addition, cervicogenic factors such as persistent neck pain and poor posture can amplify head pain through sensitization within the trigeminocervical complex (TCC), potentially increasing attack burden in susceptible individuals [83, 106]. Physical therapy and targeted musculoskeletal interventions may therefore be helpful adjuncts in appropriately selected patients.

Taken together, lifestyle and behavioral factors are clinically meaningful because they influence attack threshold, attack frequency, and overall burden. Their effects, however, are neither uniform nor equivalent to validated epidemiological risk factors for disease progression. Management should therefore emphasize individualized assessment, avoidance of overgeneralized trigger lists, and integration of behavioral strategies within a broader preventive framework [59, 72].

#### 3.2.2 Risk Factors for Chronification

Chronification refers to progression from episodic migraine to CM and should be treated as a distinct epidemiological and clinical process. Current evidence indicates that approximately 2.5–3.0% of individuals with episodic migraine progress to CM each year [57]. Among the most robust predictors, high baseline headache frequency is particularly important: patients with at least 10 headache days per month have a substantially increased risk of transition, with a pooled risk ratio (RR) of 5.95 (95% CI 4.75–7.46) [57]. This association is much stronger than that observed for most putative lifestyle triggers and supports distinguishing day‐to‐day attack modifiers from longitudinal disease‐progression factors.

Medication overuse is another major predictor of chronification. Meta‐analytic evidence indicates that medication overuse is associated with an approximately 8.8‐fold greater risk of progression to CM (95% CI 2.88–27.00) [57]. This relationship is clinically and conceptually important, because medication overuse is both a marker of high disease burden and a potentially modifiable contributor to worsening headache frequency. Likewise, depression is a reproducible predictor of progression, with a pooled RR of 1.58 (95% CI 1.35–1.85) [57]. These findings support the view that chronification reflects the interaction of headache burden, treatment behavior, and comorbid biopsychosocial vulnerability rather than exposure to isolated external triggers.

Additional factors associated with CM include anxiety, sleep disorders, obesity, metabolic disease, pain disorders, adverse childhood experiences, and head or neck injury [57]. However, not all associated factors have the same level of evidentiary support, and causal inference remains limited for many of them. For this reason, it is more accurate to refer to them as risk markers or associated progression factors unless stronger longitudinal or causal‐inference evidence is available. Overall, the key modifiable targets with the strongest present support are reduction of medication overuse, optimization of acute treatment effectiveness, management of depression and sleep disturbance, and early intervention in patients with escalating headache frequency [57].

#### 3.2.3 Socioeconomic and Health‐System Modifiers

Socioeconomic context and health‐system performance also shape migraine burden and progression, even though they are often underemphasized in biologically oriented reviews. Population studies suggest that migraine prevalence itself is not uniformly associated with socioeconomic status across all settings, but lower income, poverty, and rural dwelling are consistently linked to worse access to care, higher levels of unmet need, and greater likelihood of high‐frequency headache or CM [15]. These factors are therefore best conceptualized not simply as demographic correlates, but as modifiers of disease burden, diagnostic delay, and treatment trajectory.

Health‐system deficiencies materially contribute to poor outcomes. In many settings, migraine remains underdiagnosed, misdiagnosed, or undertreated, and preventive therapy is substantially underused even among eligible patients [15]. Such failures can indirectly promote chronification by prolonging reliance on ineffective acute treatment, encouraging medication overuse, and delaying initiation of evidence‐based prevention. The burden is particularly pronounced in low‐resource settings, where inadequate professional education, poor reimbursement, and restricted access to migraine‐specific medications further magnify disability [15]. Thus, socioeconomic disadvantage and weak systems of care should be recognized as upstream modifiers of migraine progression and burden, operating through delayed diagnosis, fragmented care, and limited treatment access rather than through direct biological mechanisms.

In summary, modifiable influences on migraine operate at multiple levels. Lifestyle and behavioral factors primarily affect attack expression; a narrower group of variables, especially high headache frequency, medication overuse, and depression, predict chronification; and socioeconomic or health‐system barriers shape whether migraine is effectively recognized and treated before progression occurs. Maintaining these distinctions improves both scientific precision and clinical usefulness.

### 3.3 Clinical Comorbidities and Shared Biology

#### 3.3.1 Psychiatric Comorbidities

Migraine is strongly associated with psychiatric comorbidity, particularly anxiety disorders, depressive disorders, and posttraumatic stress disorder (PTSD). These associations are clinically important because psychiatric comorbidity is linked to greater disability, poorer quality of life, and a higher likelihood of CM rather than merely representing coincidental co‐occurrence [71, 78, 79, 80]. Contemporary evidence also supports a bidirectional relationship for at least some of these disorders, especially depression, although the strength and direction of association can vary by study design and phenotype definition [11, 15]. The key implication for clinical practice is that psychiatric comorbidities should be regarded as integral modifiers of migraine burden and course, warranting routine recognition and management. Migraine is particularly strongly linked with depression and anxiety, and depression is also associated with increased risk of transformation from episodic to CM [15]; depression is approximately 2.5 times more common in people with migraine than in the general population, and genetic studies support substantial overlap with major depressive disorder [11].

Anxiety and depression are the most prevalent psychiatric comorbidities in migraine, with epidemiological studies consistently showing higher rates in people with migraine than in those without migraine [71, 78, 79, 80]. However, broad statements such as “2‐ to 3‐fold higher rates” should be interpreted cautiously because effect sizes vary across populations, diagnostic methods, and whether episodic and CM are analyzed separately [15]. Mechanistically, the association between migraine and anxiety or depression is likely multifactorial, involving shared genetic liability, altered central pain and salience‐network processing, stress‐system dysregulation, and possibly inflammatory signaling, but causal language should remain restrained [71, 80]. In patients with CM, psychiatric comorbidity is associated with greater functional impairment and may complicate treatment response, but evidence that it specifically reduces responsiveness to CGRP‐targeted therapies remains limited and should not be overstated [81, 107]. Pediatric migraine is likewise associated with elevated anxiety and depressive symptoms, supporting early psychosocial screening in age‐appropriate care pathways [108, 109].

PTSD is also an important psychiatric comorbidity of migraine, particularly in the context of childhood adversity and trauma exposure. Available studies support an association between childhood trauma, PTSD, and greater migraine frequency, severity, and disability [110, 111, 112]. PTSD‐related hyperarousal, stress responsivity, and trauma‐related symptom burden may amplify migraine severity and complicate self‐management, while recurrent disabling migraine may in turn worsen psychological distress [113]. Emerging genetic and epigenetic findings further suggest some biological overlap between migraine and PTSD, but these data should be presented as supportive of shared vulnerability, not as proof of a specific causal pathway [11]. On this basis, trauma‐informed care is a reasonable clinical framework for patients in whom trauma exposure or PTSD contributes substantially to symptom burden [114].

Psychiatric comorbidity has important therapeutic implications. Behavioral interventions, particularly CBT, can improve headache‐related outcomes while also addressing anxiety, depressive symptoms, maladaptive coping, and insomnia [109, 115]. By contrast, the evidence for acupuncture and nutritional interventions as dual‐purpose treatments for both migraine and psychiatric symptoms is more preliminary and should be described cautiously rather than as established practice [116, 117]. Overall, the strongest evidence supports routine screening for anxiety, depression, trauma exposure, and sleep‐related psychopathology, followed by integrated multidisciplinary management when indicated. This approach is likely to improve quality of life and may also reduce the risk of worsening headache frequency and chronicity [15, 57].

#### 3.3.2 Neurological and Pain Comorbidities

Migraine is frequently comorbid with other neurological and pain disorders, most consistently epilepsy, cerebrovascular disease, and chronic extracephalic pain syndromes. These associations are clinically important because they can increase disability, complicate diagnosis, and influence treatment selection. However, the mechanisms underlying these comorbidities are heterogeneous and should not be reduced to a single pathway. Current evidence supports contributions from altered neuronal excitability, shared genetic susceptibility, trigeminovascular dysfunction, and vascular factors; by contrast, claims regarding direct causation through neuroinflammation alone remain insufficiently established [11, 15]. Thus, this comorbidity network is best understood as reflecting partially shared biology rather than a uniform causal sequence.

Epilepsy is one of the best established neurological comorbidities of migraine, and the association appears to be bidirectional: people with migraine have a higher prevalence of epilepsy, and migraine is more common in people with epilepsy than in the general population [95]. Shared susceptibility is biologically plausible, particularly through ion‐channel and synaptic genes that regulate neuronal excitability. Variants in genes such as SCN1A and KCNMA1, as well as overlap with other channelopathy‐related genes, support this concept [11, 95]. MwA appears to show the strongest association with epilepsy, which is mechanistically consistent with cortical hyperexcitability and the role of CSD as the electrophysiological substrate of aura [90, 95]. Nevertheless, CSD should not be described as directly “causing” epileptogenesis; rather, migraine and epilepsy likely share susceptibility to abnormal excitation–inhibition balance. Clinically, this overlap is relevant because some antiseizure medications, such as valproate and topiramate, can be effective in both disorders, whereas treatment choice must still be individualized according to comorbidity profile and adverse‐effect risk.

Cerebrovascular disease is another important comorbidity of migraine, particularly in patients with MwA. Epidemiological studies consistently indicate that MwA is associated with a higher risk of ischemic stroke than MwoA or no migraine [41, 42, 118]. The statement is strongest for relative risk, whereas absolute risk in most individual patients remains low and is modified by age, smoking, hypertension, estrogen exposure, and other vascular factors [15]. The pregnancy‐associated literature also supports increased odds of stroke and TIA in women with migraine during pregnancy and the puerperium, although these estimates derive largely from observational data and should therefore be interpreted with awareness of potential confounding [41]. Migraine has also been associated with cervical artery dissection, an important cause of stroke in younger adults, and emerging genetic studies suggest shared vascular susceptibility across migraine, stroke, and cervical artery dissection [42]. However, causal inference remains unsettled, because Mendelian‐randomization analyses have not uniformly supported a direct causal effect of migraine on all stroke subtypes [11].

Neuroimaging abnormalities and long‐term cognitive outcomes require especially cautious interpretation. White‐matter hyperintensities are reported more often in some migraine cohorts, particularly in patients with aura or high attack frequency, but their pathogenic and prognostic significance remains uncertain [44, 119]. They should therefore not be presented as definitive evidence of progressive cerebrovascular injury or as established mediators of later cognitive decline. Similarly, although some studies suggest that migraine, especially MwA, may be associated with a modestly increased risk of dementia, current evidence is inconsistent and does not support a uniform conclusion that migraine leads to neurodegeneration [119, 120, 121]. Interictal deficits in attention, processing speed, and executive function have been described in some patients, but these are generally subtle and may fluctuate with attack burden, mood symptoms, sleep disturbance, and treatment status [122]. Thus, cognitive dysfunction in migraine is better viewed as a heterogeneous and often reversible clinical correlate rather than a fixed progressive consequence.

Migraine commonly coexists with neck pain, low back pain, and other chronic pain conditions, and these comorbidities are particularly frequent in CM [15, 57]. Their coexistence may reflect shared central sensitization, enhanced pain vulnerability, and reciprocal worsening of disability rather than simple anatomical overlap. In practice, pain comorbidity matters because it increases overall symptom burden, may promote medication overuse, and often necessitates broader multidisciplinary management [15, 44]. Overall, neurological and pain comorbidities should be interpreted as clinically meaningful components of migraine burden, while mechanistic claims remain calibrated to the strength of current evidence.

#### 3.3.3 Cardiovascular Risk

Migraine, particularly MwA, is associated with an increased risk of several cardiovascular and cerebrovascular outcomes, including ischemic stroke, myocardial infarction (MI), and selected vascular complications of pregnancy [41, 86]. Proposed mechanisms include endothelial dysfunction, altered vascular reactivity, prothrombotic tendencies, and shared genetic susceptibility, although the relative contribution of each remains uncertain. Genetic and epidemiological data also suggest overlap between migraine and vascular integrity pathways, but current causal‐inference studies do not uniformly support a direct causal effect of migraine on all stroke subtypes.

Ischemic stroke is the best established vascular complication of migraine, with the excess risk concentrated in patients with MwA [41, 86]. The association is generally reported as an approximately 1.5–2‐fold increase in relative risk, with the greatest relative effect observed in younger women, particularly in the presence of additional vascular risk factors such as smoking, hypertension, and estrogen exposure [15, 41, 86]. However, this relative increase should not obscure the fact that the absolute risk remains low for most individual patients. MwA is an independent vascular risk factor, and its clinical significance is amplified by coexisting conventional risk factors [87]. Likewise, although patients with CM may exhibit a higher burden of vascular comorbidity, this does not establish that migraine chronification itself is a familial vascular syndrome [123]. In older adults, where vascular events and risk factors increase regardless of migraine status, a careful cardiovascular history remains essential; notably, MwA is recognized as a risk factor for MI, stroke, and all‐cause cardiovascular mortality [44].

The association between migraine and MI is weaker and less specific than that for ischemic stroke, but population‐based studies do support a modest increase in risk, again more consistently in MwA than in MwoA [86, 87]. Mechanistic explanations should be stated conservatively. Endothelial dysfunction and impaired vascular homeostasis are plausible shared mechanisms, and genetic studies support links between migraine and vascular integrity pathways [11]. However, surrogate cardiac electrophysiological findings such as prolonged P‐wave dispersion or atrial electromechanical delay [124] should not be overinterpreted as established mechanistic bridges between migraine and ischemic heart disease. Similarly, oxidative stress and mitochondrial dysfunction may contribute to migraine biology and vascular vulnerability, but their direct role in explaining excess MI risk in migraineurs remains incompletely defined [125]. Thus, the most defensible conclusion is that migraine, particularly MwA, clusters with vascular risk through partially shared biology and risk‐factor aggregation rather than through a single validated mechanism.

Pregnancy represents a special clinical context in which migraine has important vascular implications. Observational studies indicate that migraine during pregnancy is associated with an increased risk of hypertensive disorders of pregnancy, including gestational hypertension and preeclampsia, and may also be associated with stroke and thromboembolic complications [41, 126, 127]. The evidence is strongest for an association rather than causation, and estimates may be influenced by confounding from age, obesity, and baseline vascular risk [126, 127]. A contemporary clinical review likewise notes that migraine is associated with hypertensive disorders of pregnancy, including preeclampsia, with higher risk reported in women older than 30 years and in those with obesity [128]. Therefore, in pregnant patients with migraine—especially those with MwA or coexisting vascular risk factors—closer blood pressure surveillance and obstetric risk assessment are justified.

Overall, cardiovascular risk in migraine should be interpreted within a risk‐stratification framework. MwA is the phenotype most consistently associated with ischemic stroke and other major vascular outcomes; risk is modified by age, sex, smoking, blood pressure, lipid status, and hormonal exposures; and treatment choice should account for both migraine severity and vascular comorbidity.

#### 3.3.4 Sleep, Metabolic, and Other Systemic Comorbidities

Migraine is associated with a broad range of sleep‐related, metabolic, vestibular, gastrointestinal, and other systemic comorbidities that can amplify symptom burden and complicate management. These associations are clinically relevant, but they should not be presented as reflecting a single shared mechanism. Current evidence supports partially overlapping contributions from altered hypothalamic and brainstem regulation, trigeminovascular activation, neuropeptide signaling, autonomic dysfunction, and shared genetic susceptibility, whereas more direct mechanistic claims—particularly those invoking neuroinflammation as a unifying explanation—should remain cautious [11, 98]. In epidemiological and clinical studies, many of these comorbidities are more frequent in CM than in episodic migraine, suggesting that they may function as burden amplifiers and, in some cases, progression‐associated factors rather than merely coincidental coexisting conditions [39, 57, 82]. CM is associated with an increased likelihood of sleep disorders, obesity, metabolic disease, and asthma, supporting their relevance as systemic comorbidities rather than isolated findings [57].

Sleep disorders are among the most important systemic comorbidities of migraine. Insomnia, sleep deprivation, and irregular sleep–wake patterns are consistently associated with greater migraine frequency and severity, and the relationship appears bidirectional: poor sleep can lower the threshold for attacks, whereas migraine itself can disrupt sleep continuity [39, 70, 82]. Recent genetic studies strengthen this association by suggesting that genetically predicted insomnia contributes to increased migraine risk, whereas evidence for reverse causality is weaker [11]. Obstructive sleep apnea is also clinically relevant, particularly because it may worsen headache burden through sleep fragmentation, intermittent hypoxemia, and increased cardiometabolic stress; however, direct mechanistic statements linking sleep apnea to migraine through neuroinflammation alone are too simplistic [39, 70, 82]. Thus, sleep comorbidity should be framed as an important modifier of migraine burden and treatment response rather than as a uniform causal pathway.

Metabolic and gastrointestinal comorbidities are also increasingly recognized. Obesity and metabolic disease are more common in CM and may contribute to higher headache frequency, although causal relationships remain incompletely resolved [57, 82]. Genetic correlation and Mendelian‐randomization studies further support links between migraine and traits such as blood pressure, Type 2 diabetes, lipids, and sleep‐related phenotypes, indicating that at least part of this overlap reflects shared biology [11]. Gastrointestinal symptoms—including nausea and vomiting—are core features of migraine attacks and are associated with greater symptom severity and disability [40, 129]. Delayed gastric emptying or impaired gastrointestinal motility may reduce absorption of oral acute medications, which is clinically important even when formal gastroparesis is not established [39, 40, 44]. In addition, irritable bowel syndrome appears to be more common in migraine, and recent Mendelian‐randomization data support IBS as a possible contributor to migraine risk rather than a purely coincidental association [11, 84]. By contrast, mechanistic claims that trigeminovascular innervation of the gastrointestinal tract alone explains these comorbidities are overly reductionist and should be qualified [85].

**FIGURE 2:**
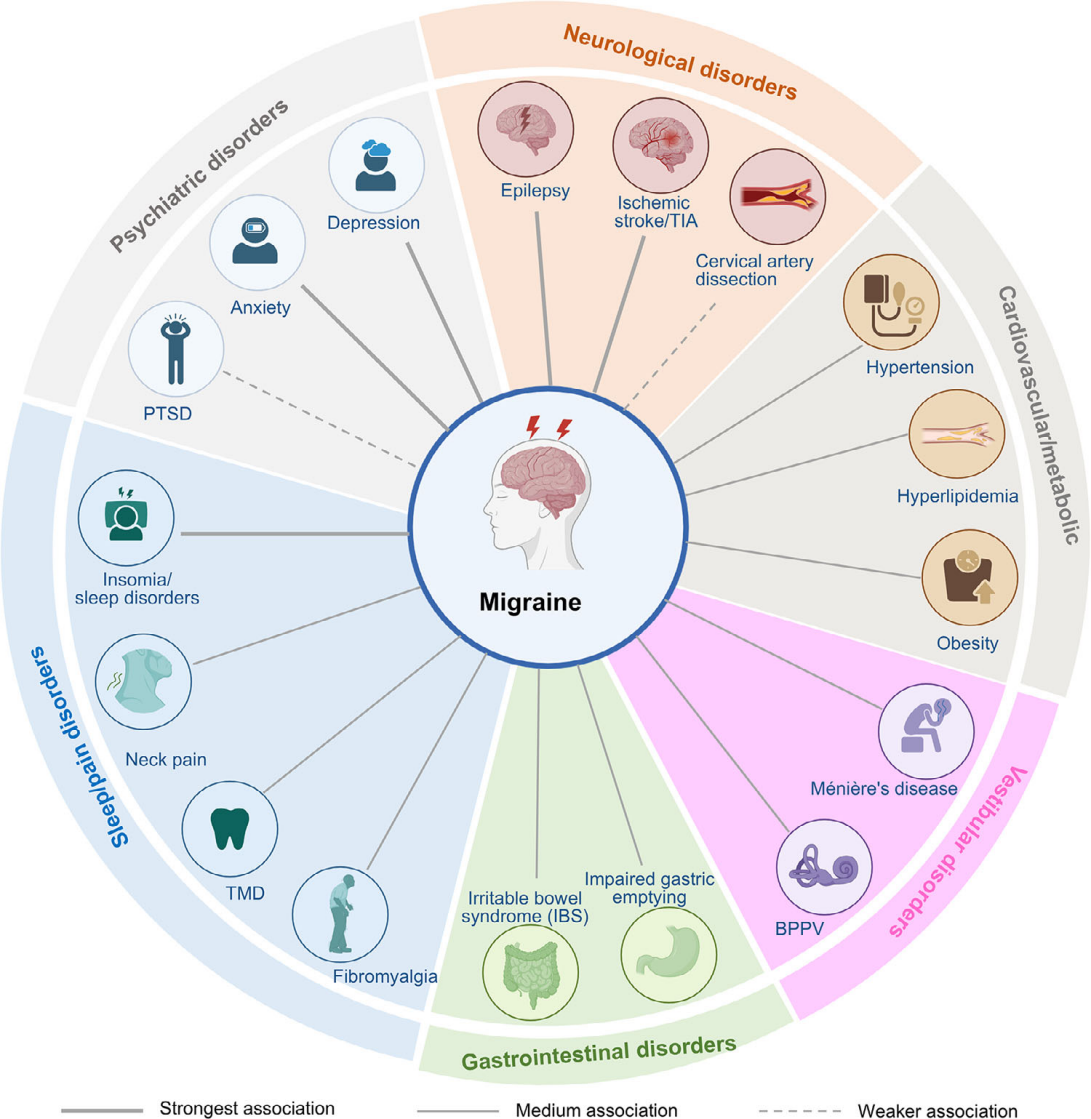
Clinical comorbidity network of migraine. The circular network diagram places migraine at the center and maps its major associated conditions across psychiatric, neurological, vascular, vestibular, gastrointestinal, sleep, and chronic pain domains. The strongest and most clinically relevant links highlighted in this review include depression, anxiety, epilepsy, sleep disorders, chronic pain syndromes (fibromyalgia, TMD, neck pain), and migraine with aura‐associated cerebrovascular risk (ischemic stroke/TIA, cervical artery dissection). Vestibular and gastrointestinal associations, including Ménière's disease, benign paroxysmal positional vertigo, irritable bowel syndrome, and impaired gastric emptying, are shown as additional burden‐amplifying comorbidities. BPPV, benign paroxysmal positional vertigo; IBS, irritable bowel syndrome, PTSD, posttraumatic stress disorder; TIA, transient ischemic attack; TMD, temporomandibular disorder. Illustrations were created via biorender (http://app.biorender.com).

Vestibular and other pain‐related systemic associations also merit attention. Vestibular migraine is the most common vestibular disorder linked to migraine and is characterized by episodic vertigo, dizziness, or imbalance occurring in a migraine context [130, 131, 132]. Although vestibular migraine often coexists with anxiety, depression, and sleep disturbance, these accompanying disorders should be viewed as burden modifiers rather than defining features [40, 132]. Migraine has also been associated epidemiologically with Ménière's disease and benign paroxysmal positional vertigo, but shared mechanisms remain incompletely understood and should not be reduced to established roles for endolymphatic hydrops or CGRP dysregulation alone [130, 131, 133]. Likewise, TMD and neck pain are more prevalent in people with migraine and may exacerbate headache through shared pain vulnerability and trigeminocervical sensitization rather than through a simple peripheral trigger model [84, 85]. Overall, these sleep, metabolic, vestibular, gastrointestinal, and pain‐related comorbidities reinforce that migraine is a multisystem disorder whose optimal management often requires recognition and treatment of coexisting conditions rather than headache‐focused therapy alone (Figure 2).

## 4 Pathophysiology of Migraine

Migraine pathophysiology is best understood as a dynamic, phase‐dependent process involving distributed neural, vascular, immune, and glial mechanisms rather than a single vascular or inflammatory event [128, 134, 135]. This section first summarizes the conceptual evolution from classical vascular theories to contemporary neurovascular and network‐based models. It then reviews key biological processes implicated in migraine generation and progression, including CSD, trigeminovascular activation, neuropeptide signaling, neurogenic inflammation, central sensitization, and glial–neuronal interactions. Finally, these mechanisms are integrated across the clinical phases of migraine, from premonitory symptoms and aura to headache, postdrome, and chronification, to provide a coherent framework linking biological mechanisms with clinical manifestations and therapeutic targets.

### 4.1 From Neurovascular Theory to a Distributed Brain Network Model

Historically, migraine was conceptualized primarily as a vascular disorder in which headache was attributed to abnormal cranial vasodilation. Although vascular changes remain biologically relevant, this formulation is now too narrow. Current evidence supports migraine as a distributed brain network disorder that engages both central and peripheral mechanisms, with the trigeminovascular system providing the principal substrate for headache generation [128, 136]. In this contemporary model, nociceptive input arises from first‐order trigeminal afferents innervating the meninges and intracranial vessels, converges in the TCC, and is then relayed to thalamic and cortical regions that generate the sensory‐discriminative, autonomic, affective, and cognitive features of the attack [128, 137]. This framework explains why migraine cannot be reduced to a simple disorder of vasomotor tone. Rather, it reflects altered sensory processing and network excitability that simultaneously engage vascular, neural, and neuropeptidergic systems.

Within this framework, the trigeminovascular system remains central to the pain phase of migraine. Peripheral trigeminal afferents arising from the trigeminal ganglion (TG) innervate pain‐sensitive intracranial structures, especially the dura mater and its associated vessels, and project centrally to second‐order neurons in the TCC [128, 137]. These neurons then ascend to the thalamus, where nociceptive information is integrated and relayed onward to cortical regions involved in somatosensory, insular, visual, autonomic, and limbic processing [128, 137]. The thalamus is therefore not merely a passive relay, but a key hub linking trigeminovascular input to symptoms such as photophobia, allodynia, and altered sensory salience [128]. In parallel, brainstem and diencephalic regions—including the periaqueductal gray, locus coeruleus, rostral ventromedial medulla, pontine regions, and hypothalamus—modulate trigeminovascular transmission and likely contribute to sensory amplification, autonomic symptoms, and attack initiation [128]. Notably, neuroimaging studies have identified hypothalamic activation and altered hypothalamic‐brainstem connectivity during the premonitory phase, even before headache onset, reinforcing the view that migraine is not solely a peripheral nociceptive disorder but also involves early dysfunction in central homeostatic networks [3, 128].

At the molecular level, CGRP is a major effector of migraine biology, but it should not be presented as a complete mechanistic explanation. CGRP is released from trigeminal afferents during attacks, promotes potent vasodilatory and nociceptive signaling, and modulates both peripheral and central pain transmission [128, 136, 138]. Its clinical importance is strongly supported by provocation studies and by the efficacy of CGRP‐targeted therapies [138, 139, 140]. Pituitary adenylate cyclase‐activating polypeptide (PACAP) is another important neuropeptide within the trigeminovascular network and, like CGRP, can provoke migraine in susceptible individuals [139, 140]. Recent human data further indicate that PACAP signaling is at least partly parallel to the CGRP pathway: pretreatment with eptinezumab did not prevent PACAP38‐induced migraine, and a Phase 2 trial of a PACAP‐ligand monoclonal antibody showed preventive efficacy [140, 141]. TRPM3 channels, which are expressed in trigeminovascular tissues, have also emerged as candidate modulators of sex‐related differences in trigeminovascular excitability and may interact with CGRP signaling, potentially contributing to the higher prevalence and severity of migraine in females [101]. Together, these mediators support a neuropeptidergic model in which migraine pain arises from activation and sensitization of trigeminovascular pathways rather than from vasodilation alone.

The vascular component of migraine should also be interpreted with appropriate caution. Neuroimaging studies showing altered neurovascular coupling (NVC), regional cerebral blood flow, or ocular microvascular measures suggest dysregulation of neurovascular responses, but they do not establish a purely vascular origin of migraine [142, 143, 144, 145]. In particular, abnormal NVC may reflect altered cortical excitability or metabolic demand rather than a primary vascular lesion. At the same time, recent magnetic resonance angiography data indicate that several molecular migraine triggers consistently dilate extracerebral rather than intracerebral arteries, supporting a relevant peripheral trigeminovascular component to headache expression without reinstating a purely vascular theory [146]. Retinal and cerebrovascular imaging abnormalities may therefore be better interpreted as correlates of altered neurovascular physiology than as evidence of a primary vascular lesion. The original discussion of neurovascular compression in ophthalmoplegic migraine is not representative of migraine pathophysiology more broadly and should not be used to support the general neurovascular model, especially because recurrent painful ophthalmoplegic neuropathy is now classified separately from primary migraine in modern headache nosology [137].

Peripheral trigeminal activation can be accompanied by release of vasoactive and inflammatory mediators, and this signaling may contribute to nociceptor sensitization. However, direct evidence that dural inflammation is the primary generator of migraine pain remains limited, and peripheral neurogenic inflammation should not be presented as a complete explanation of migraine headache [128]. Contemporary models instead favor a multilevel process in which central network dysfunction, trigeminovascular activation, neuropeptide release, and peripheral as well as central sensitization interact dynamically across the migraine attack [128, 136, 137]. This broader formulation is more consistent with current mechanistic and translational evidence than the traditional vascular theory alone.

### 4.2 CSD: Aura Mechanism and Link to Headache

CSD is the best‐supported electrophysiological substrate of migraine aura. It is defined as a slowly propagating wave of near‐complete neuronal and glial depolarization that transiently collapses transmembrane ion gradients, suppresses spontaneous and evoked cortical activity in its wake, and produces reversible metabolic and hemodynamic perturbations [147, 148]. The term “cortical spreading depolarization” is preferable to “cortical spreading depression” alone because the initiating event is the depolarization itself, whereas the subsequent depression of electrical activity is secondary [148]. Clinically, CSD provides the most coherent explanation for the gradual onset, orderly spread, and reversibility of aura symptoms, particularly visual aura, the most common phenotype [147, 148]. Direct intracranial and electrocorticographic observations in humans, together with convergent hemodynamic and neuroimaging data, strongly support CSD as the biological correlate of migraine aura [147, 148, 149, 150].

The initiation and propagation of CSD depend on a critical threshold of cortical excitability. Once triggered, CSD is sustained by a marked extracellular rise in K+ and widespread release of excitatory neurotransmitters, especially glutamate, with propagation strongly facilitated by NMDA receptor activation [148, 151, 152]. The process also involves substantial Ca^2+^ influx, osmotic water shifts, and transient tissue swelling, reflecting the intense metabolic burden imposed on the cortex [148]. Genetic data further support this framework: mutations in FHM genes increase susceptibility to CSD by disrupting ion homeostasis and excitatory‐inhibitory balance [152]. In particular, variants affecting CACNA1A, ATP1A2, and SCN1A converge on enhanced glutamatergic transmission, impaired potassium clearance, and cortical hyperexcitability, thereby lowering the threshold for CSD [152, 153].

CSD is strongly linked to aura, but its relationship to headache requires more careful formulation. Current evidence indicates that CSD can activate the trigeminovascular system, trigger delayed meningeal nociceptor firing and induce trigeminal pain behavior in preclinical models [148, 154, 155, 156].

A more direct biological bridge between cortex and peripheral trigeminal structures has recently been proposed: cerebrospinal fluid (CSF) can reach the TG, and after CSD the CSF proteome is altered such that CSF collected from CSD‐exposed animals activates TG neurons in naïve animals, in part via CSF‐borne CGRP [157]. This mechanism substantially strengthens the plausibility that a cortical event can directly influence peripheral trigeminal neurons without requiring a purely vascular or purely synaptic relay. Accordingly, CSD is best viewed as the established mechanism of aura and an increasingly well‐supported trigger of headache in MwA, although direct human demonstration of aura‐to‐headache causality in individual attacks remains limited [148, 157]. Aura can occur without headache, and many migraine attacks occur without clinically apparent aura [148].

The hemodynamic consequences of CSD are complex and should not be reduced to a simple vasoconstriction‐then‐vasodilation sequence. Experimental studies show species‐ and model‐dependent patterns that may include transient hyperemia, hypoperfusion, and more prolonged oligemia, whereas in humans the dominant hemodynamic signature of aura is spreading oligemia that does not respect vascular territories [148, 149, 158]. This observation argues against the older vasospastic interpretation of aura. CSD‐associated blood flow changes are now better understood as components of a neurovascular‐metabolic response to intense depolarization rather than the primary cause of aura symptoms [148]. Similarly, although blood–brain barrier alterations and vascular permeability changes have been described in preclinical models, evidence for clinically meaningful BBB disruption during typical migraine attacks in humans remains limited and method dependent [148, 159].

Glial cells, especially astrocytes and microglia, are increasingly recognized as important modulators of CSD and its downstream consequences. Astrocytes regulate extracellular K+ and glutamate, shape local vascular responses, and influence susceptibility to CSD; the strong association of ATP1A2 mutations with FHM Type 2 underscores the importance of astrocytic ion and glutamate clearance in this process [11, 134, 160]. Current evidence suggests that astrocytes are particularly important for setting the initiation threshold of CSD and for shaping its vascular and metabolic consequences, rather than serving as the sole drivers of propagation [134, 160]. Microglia are also activated by CSD and may contribute to downstream inflammatory amplification, particularly in repeated depolarization paradigms, but the evidence remains substantially stronger in preclinical models than in human in vivo studies [134, 156, 160]. Accordingly, glial involvement should be presented as an important and evolving mechanistic layer rather than as a fully resolved causal pathway in patients.

One influential mechanistic line of work showed that CSD opens neuronal pannexin‐1 (Panx1) channels and is followed by inflammasome‐related signaling, Caspase‐1 activation, and HMGB1 release from neurons, which in turn activates NF‐κB signaling in astrocytes [161]. Suppression of this cascade abolishes CSD‐induced trigeminovascular activation, dural mast‐cell degranulation, and headache‐related behavior in experimental models, supporting a model in which neuronal stress signaling propagates from cortex to pain‐sensitive meningeal and trigeminal structures [161]. More recent work places this pathway within a broader framework of transient parenchymal‐to‐meningeal neuroimmune signaling, in which CSD induces release of ATP, HMGB1, nitric oxide, cytokine‐related signals, and inflammasome‐associated mediators that can sensitize trigeminovascular afferents [154, 159, 160, 162]. The roles of Panx1, P2×7 signaling, and inflammasome‐related pathways have become central to current mechanistic models [161, 162]. Recent cell‐specific analyses further suggest that after a single CSD event, early astrocytic proinflammatory signaling is followed by a more delayed anti‐inflammatory/resolution profile, whereas more repetitive paradigms are more likely to drive robust microglial activation and a stronger proinflammatory phenotype [156, 163]. This distinction helps avoid overgeneralizing findings from repetitive experimental paradigms to typical human aura and supports describing the process as transient neuroimmune signaling rather than diffuse or sustained inflammatory injury.

Finally, CSD has important translational relevance. Preventive agents can reduce susceptibility to spreading depolarization in experimental systems, and therapies that inhibit the trigeminovascular consequences of CSD can reduce headache‐related behavior without necessarily suppressing CSD propagation itself [148, 151, 164, 165]. This distinction is mechanistically important: current evidence suggests that CGRP‐pathway therapies act predominantly on downstream nociceptive and trigeminovascular signaling rather than on aura generation itself [148]. Emerging neuromodulatory approaches, including vagus nerve stimulation, may also modulate CSD susceptibility, but most supporting evidence remains preclinical and should be interpreted cautiously [164, 165]. Overall, CSD should be presented as a central mechanism of migraine aura, a biologically plausible and increasingly well‐supported trigger of headache in MwA, and a useful translational model for mechanism‐based therapeutic development, while acknowledging that direct human electrophysiological data remain limited and that aura‐specific treatments are still underdeveloped [148, 150, 166].

### 4.3 Genetic and Molecular Mechanisms

Migraine is a genetically complex neurobiological disorder in which rare, high‐impact variants and common, small‐effect susceptibility alleles converge on a limited set of biological processes, particularly neuronal excitability, glutamatergic neurotransmission, ion homeostasis, vascular regulation, and neuropeptide signaling [11, 60, 167, 168]. The earlier view that migraine genetics is explained mainly by ion‐channel dysfunction is now incomplete. Contemporary genomic data instead support a continuum from monogenic to polygenic disease, with shared mechanistic themes across these forms [11]. Heritability is estimated at approximately 30–60%, and MwA appears to be more heritable than MwoA, consistent with stronger genetic loading in aura‐related biology [11].

The clearest mechanistic insights come from FHM and related Mendelian syndromes. The canonical FHM genes—CACNA1A, ATP1A2, and SCN1A—encode proteins that regulate synaptic transmission and excitatory–inhibitory balance at glutamatergic and GABAergic synapses [11, 169, 170]. Although these genes are often grouped under the label of “ion‐channel disorders,” their biological effects extend well beyond channel gating alone. Gain‐of‐function variants in *CACNA1A* increase presynaptic Ca^2+^ influx and enhance glutamatergic neurotransmission; loss‐of‐function variants in *ATP1A2* impair astrocytic clearance of extracellular K^+^ and glutamate; and FHM‐associated SCN1A variants, typically gain‐of‐function, perturb interneuronal excitability and destabilize inhibitory network control [11, 169, 170]. These convergent effects lower the threshold for CSD and reinforce the concept that migraine, particularly aura‐prone migraine, arises from disordered neuroglial homeostasis rather than from a purely vascular lesion [11]. Additional genes implicated in hemiplegic or familial migraine—including *PRRT2*, *SLC1A3*, *SLC2A1*, *SLC4A4*, *KCNK18*, and *CSNK1D—*further support roles for synaptic vesicle release, astrocytic glutamate transport, energy supply, pH regulation, trigeminal excitability, and circadian–neuronal interactions [11].

For common migraine, the genetic architecture is predominantly polygenic. Large GWAS meta‐analyses have now identified over 120 susceptibility loci, and subsequent sequencing‐augmented and fine‐mapping analyses have further refined these associations, including the identification of 181 credible sets across 102 risk regions and the prioritization of likely causal variants such as missense variants in NGF and INPP5A [4, 11, 60, 167]. Importantly, GWAS signals are enriched in genes expressed in both CNS and vascular cell types, supporting a combined neuronal–vascular framework rather than a purely neuronal or purely vascular model [11]. Fine‐mapping and expression‐based analyses group these loci into functional domains that include synaptic function and neurotransmitter regulation, ion‐channel function, calcium homeostasis, extracellular matrix and vascular integrity, vascular development and maintenance, nociception, metabolism and homeostasis, and inflammation [11]. Thus, common migraine genetics does not simply reinforce the classic neurovascular theory; rather, it supports a broader model in which susceptibility arises from perturbations across interconnected neuronal, glial, vascular, and metabolic systems.

At the molecular level, glutamatergic signaling remains one of the most coherent mechanistic themes linking monogenic and polygenic migraine. FHM1 and FHM2 mutations converge on increased extracellular glutamate and facilitation of CSD [11, 169, 170]. Variants in SLC1A3 and related transport systems further support the importance of impaired glutamate reuptake in migraine biology [11]. Together, these findings indicate that altered glutamate handling can influence cortical excitability, synaptic spillover, and susceptibility to CSD, particularly when astrocytic buffering is compromised [11]. Experimental and genetic data strongly support glutamatergic dysregulation, whereas direct therapeutic targeting of this pathway in migraine has so far yielded mixed and limited clinical benefit. Serotonergic signaling is also relevant, particularly in attack modulation and in the pharmacology of triptans, but current migraine genetics does not support a simple serotonin‐deficiency model [128, 171, 172]. CGRP can provoke migraine‐like headache in susceptible individuals; although aura can also be induced in a subset of patients with MwA, the evidence remains less robust than for headache provocation and does not establish CGRP as a general mechanism of aura generation [128, 173, 174, 175, 176]. This distinction is important because it avoids conflating aura biology, which is most closely linked to CSD, with headache biology, in which CGRP has a central role. PACAP is also increasingly recognized as a relevant neuropeptide; recent clinical and mechanistic data suggest that it represents an additional candidate therapeutic axis that is distinct from, but partly overlapping with, the CGRP pathway [128, 140].

Differential expression of immune‐associated genes, including PIAS and SOCS family members, may indicate involvement of cytokine‐regulatory pathways in migraine susceptibility [177, 178]. However, these findings should not be taken to mean that migraine is primarily an immune disorder. Rather, immune signaling appears to intersect with neuronal excitability, glial activation, vascular regulation, and sensitization, and may be particularly relevant in certain subtypes or in chronification [11]. Similarly, reports of altered long noncoding RNAs and calcium‐signaling genes are intriguing, but they remain largely descriptive unless linked to validated functional effects [179]. These observations are therefore best interpreted as evidence of a complex regulatory architecture rather than as established pathogenic pathways on the same footing as FHM genes or CGRP biology.

Genetic overlap with other disorders provides additional insight into migraine mechanisms. Shared genetic architecture has been reported with depression, hypertension, vascular traits, epilepsy, and other neurological or systemic phenotypes, supporting pleiotropic effects across pain, vascular, and excitability networks [11, 180, 181, 182]. However, such overlap should not be overstated as evidence of direct causality. Some cross‐trait relationships likely reflect shared upstream biology rather than disease‐specific pathways. Rare hereditary small‐vessel disorders such as CADASIL, caused by NOTCH3 mutations, further underscore the intersection of vascular and neuronal mechanisms, because migraine—often with aura—can precede overt stroke or cognitive symptoms by years [11, 183, 184]. Even in this setting, however, the implication is not that ordinary migraine is simply a mild vasculopathy, but rather that vascular integrity pathways can modulate migraine susceptibility in selected genetic contexts.

Overall, current evidence supports a revised model in which migraine genetics converges on a limited number of mechanistic domains: synaptic transmission, excitatory–inhibitory balance, astrocytic ion and glutamate buffering, trigeminal nociceptive signaling, vascular maintenance, and neuropeptide‐mediated pain transmission [11, 128, 173, 174, 175, 176]. The key translational implication is therefore not merely that migraine is “genetic,” but that genetic studies have clarified why migraine is a disorder of network excitability and neurovascular–neuroimmune interaction, while also sharpening biological stratification and target discovery; the most clinically validated therapeutic pathway remains CGRP, with PACAP emerging as an additional candidate pathway [4, 140].

### 4.4 Neuroinflammation and Glial Cell Dynamics

Neuroinflammation in migraine is better conceptualized as a dynamic, spatially restricted, and phase‐dependent process than as a uniform, continuously active inflammatory state. This distinction matters because most mechanistic evidence derives from CSD, nitroglycerin, inflammatory soup, or trigeminal sensitization models, whereas direct evidence in humans remains more limited and appears to vary across migraine subtypes and attack phases [135, 185]. Current human data support glial‐related neuroimmune activity in selected contexts—notably MwA and CM—including elevated TSPO‐PET signal in nociceptive and visual regions in MA, increased [11C]PBR28 PET signal in CM [186, 187], trigeminal nerve root abnormalities linked to local neuroinflammatory signal [188], and elevated CSF CX3CL1 that appears to persist irrespective of headache status [189]. However, these observationsdo not establish a uniform degree of neuroinflammation across all patients with migraine, especially during pain‐free intervals or in MwoA, where biomarker findings remain more variable [189, 190, 191]. Taken together, the available evidence supports a model of context‐dependent glial activation, strongest during aura‐related cortical events and in chronic trigeminovascular sensitization, rather than a single inflammatory signature applicable to all migraine phenotypes.

Within this framework, astrocytes and microglia are best viewed as the principal central amplifiers of migraine‐relevant neuroinflammatory signaling. Astrocytes occupy a strategic position at the neurovascular interface and are therefore well placed to couple ionic disequilibrium, glutamatergic excess, and BBB stress to inflammatory transcriptional programs [135, 192]. In the migraine setting, CSD‐induced opening of neuronal Panx1 channels, inflammasome activation, and release of HMGB1 and IL‐1β provide a plausible upstream trigger for astrocytic NF‐κB activation [74, 161, 193]. Astrocytes can then reinforce a feed‐forward loop through impaired glutamate and K+ clearance, cytokine release, and secondary activation or recruitment of microglia [194, 195, 196, 197]. In preclinical migraine models, microglia in turn translate ATP, danger signals, and cytokine cues into purinergic, TLR, chemokine, and inflammasome signaling pathways—including P2×4/P2×7, P2Y12/P2Y14, TLR2/TLR4, and NLRP3—thereby promoting central sensitization in the TCC [198, 199, 200, 201, 202, 203, 204, 205, 206, 207, 208]. Importantly, this central glial crosstalk is not merely additive. Microglial IL‐18, inflammasome‐linked signaling, and C1q can drive reactive astrocyte states [209, 210, 211], whereas astrocytes can reciprocally shape microglial activation through ATP, IL‐33, and TGF‐β‐related mechanisms [212, 213]. Neuroinflammation in migraine is therefore better understood as a multicellular signaling network than as isolated activation of a single glial population.

Peripheral glia extends this inflammatory network beyond the brain parenchyma and help explain how neuropeptide signaling is converted into persistent nociceptor sensitization. In the TG, SGCs are activated by neuronal ATP and CGRP, upregulate purinergic signaling, and release IL‐1β, IL‐6, and TNF‐α, thereby fostering a local inflammatory microenvironment that reinforces neuronal excitability [214, 215, 216, 217, 218]. In rodent models, Schwann cells appear to play a parallel role at peripheral trigeminal endings, where CGRP receptor activation induces NO production, gates TRPA1, promotes ROS generation, and sustains periorbital mechanical allodynia [219, 220]. These peripheral mechanisms are highly relevant to migraine because they position glia not as passive bystanders, but as active transducers of trigeminal neuropeptide signaling into inflammatory amplification.

Migraine is approximately three times more prevalent in women than in men [221], and recent human data indicate that hormonal state modulates CGRP biology: women with episodic migraine and a regular menstrual cycle show higher CGRP concentrations during menstruation than matched controls, supporting the concept that estradiol withdrawal or fluctuation can potentiate CGRP‐dependent vulnerability [222]. Mechanistically, this suggests that neuron‐SGC and neuron‐Schwann cell CGRP loops are unlikely to operate independently of hormonal state, and that glial responses to CGRP signaling in the TG should be interpreted in the context of endocrine status rather than assumed to be identical across patients. A similar caution applies to the interpretation of microglial signaling pathways. Recent work identifies TLR2/4, P2×4, P2Y12/14, and NLRP3 as recurrent nodes in migraine‐related sensitization [198, 199, 200, 201, 202, 203, 204, 205, 206, 207, 208]. These are highly plausible mechanisms, but the broader pain literature indicates that the contribution of microglia to hypersensitivity can be sex dependent. Foundational work showed that microglia‐dependent pain signaling is more prominent in males, whereas females can rely more strongly on adaptive immune mechanisms, particularly T cells [223]. Subsequent work refined this view by showing that myeloid‐lineage TLR4 contributes more robustly to the transition to chronic pain in males, with weaker or qualitatively different effects in females [224]. These findings do not negate the relevance of microglia in female migraine biology; rather, they caution against assuming that microglial inhibitors, or blockade of pathways such as TLR4 or P2×4, will have identical efficacy by sex. This issue is especially important in migraine, a disorder with marked female predominance, and should temper direct translational extrapolation from predominantly male preclinical paradigms.

Astrocytes also exhibit sex‐specific regulation relevant to migraine, although this area remains underdeveloped in the current literature. Astrocytes express estrogen‐ and progesterone‐responsive signaling machinery, and recent work indicates that astrocytic estrogen receptor‐β can regulate glial activation and metabolic gene programs in a cell‐specific manner [225]. In addition, human astrocytes studied in vitro show sex‐specific metabolic and oxidative responses under inflammatory‐metabolic stress, including differences in antioxidant programs and steroid‐related signaling components [226]. These observations are not migraine specific, but they are highly pertinent to a disease in which astrocytic control of glutamate handling, energy metabolism, and inflammatory reactivity is already central [160]. They suggest that astrocyte responses to CSD, oxidative stress, and cytokine signaling may differ between females and males, with potential implications for both attack susceptibility and therapeutic response.

A phase‐resolved view helps organize these glial mechanisms along the temporal arc of a migraine attack. In the premonitory phase, direct evidence for glial activation remains limited, but glia may already contribute through altered hypothalamic‐brainstem network tone, astrocytic metabolic support, and basal microglial priming that lowers the threshold for subsequent trigeminovascular recruitment [134, 227]. In patients with aura, the clearest mechanistic entry point is CSD, which provides a temporally defined cortical event capable of initiating astrocytic and microglial inflammatory signaling through HMGB1, IL‐1β, ATP, and ionic disequilibrium [74, 193, 199, 204]. This makes the aura‐to‐early‐ictal transition the phase in which evidence for central glial participation is currently strongest. By contrast, in MO, where CSD is not clinically manifest, glial activation may depend more on subcortical sensory dysregulation, peripheral trigeminal neuropeptide release, and recurrent sensitization, but this remains less directly resolved.

During the headache phase, peripheral glia in the TG and the meningeal afferent environment likely become more prominent. SGCs and Schwann cells are well positioned to respond to ATP, CGRP, substance P, PACAP, and inflammatory mediators, thereby reinforcing peripheral sensitization [214, 215, 216, 219, 220]. Central astrocyte–microglia crosstalk within the TNC/TCC then contributes to amplification and maintenance of nociceptive transmission [209, 212, 213]. In the postdrome, direct evidence is again sparse, but persistent glial activation provides a plausible substrate for fatigue, cognitive fog, and residual sensory hypersensitivity [228, 229, 230]. This interpretation is also consistent with evidence that proresolving or anti‐inflammatory microglial programs can modulate recovery from migraine‐related neuroinflammation. This temporal framing has translational implications: interventions targeting CSD‐linked astrocytic or microglial signaling may be most relevant early in attacks or in MA, whereas therapies interrupting CGRP‐dependent peripheral glial loops may be more relevant during the headache phase. Conversely, treatments that promote glial resolution phenotypes may be better suited to limiting postattack persistence or chronification.

Finally, glial heterogeneity should be explicitly acknowledged. Much of the migraine literature still treats astrocytes, microglia, Schwann cells, and SGCs as relatively uniform populations, yet single‐cell studies show that migraine‐relevant tissues contain multiple neuronal and non‐neuronal subtypes with distinct transcriptional identities [231, 232]. Recent integrative analyses further suggest that MA and MO may differ not only clinically but also in their cellular and spatial molecular architecture [233]. This heterogeneity matters because “glial activation” is unlikely to represent a single conserved state across the cortex, TNC/TCC, meninges, and TG. Instead, regional specialization, phase specificity, sex, and disease subtype likely determine which glial programs are engaged and whether they are pathogenic, compensatory, or proresolving. Recent single‐nucleus transcriptomic work in CM models further refines this view by showing that glial populations are highly heterogeneous and trajectory dependent, rather than homogeneous “activated” pools [234]. Future studies should therefore move beyond global markers such as GFAP or Iba1 and instead define cell states with spatial and temporal precision.

**FIGURE 3:**
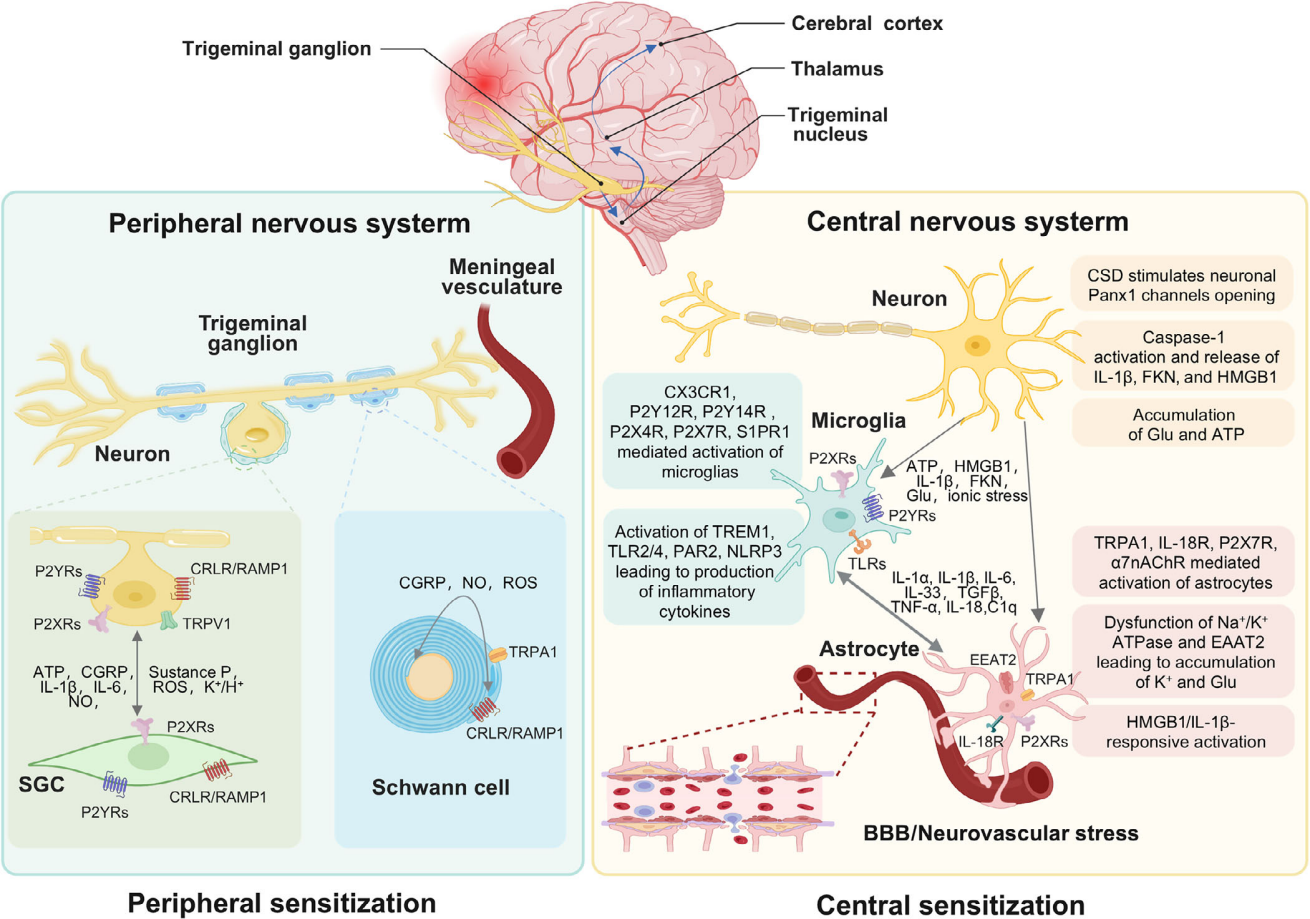
Glial heterogeneity and neuroimmune interaction network in migraine. The schematic illustrates central and peripheral glial crosstalk across migraine‐relevant compartments. In the central compartment, neuronal stress signals associated with cortical spreading depolarization and sensitization activate astrocytes and microglia, promoting NF‐kB‐linked inflammatory signaling, impaired glutamate and potassium buffering, cytokine release, and amplification of central nociceptive transmission. In the trigeminal ganglion, neuronal ATP and CGRP activate satellite glial cells, which release IL‐1beta, IL‐6, and TNF‐alpha and reinforce peripheral neuronal hyperexcitability. Together, these interactions converge on peripheral sensitization, central sensitization, and neurovascular/barrier dysfunction. TCC, trigeminocervical complex; BBB, blood–brain barrier; CSD, cortical spreading depolarization; SGC, satellite glial cell; ATP, adenosine triphosphate; CGRP, calcitonin gene‐related peptide; NO, nitric oxide; ROS, reactive oxygen species; Glu, glutamate; IL‐1α, interleukin‐1 alpha; IL‐1β, interleukin‐1 beta; IL‐6, interleukin‐6; IL‐18, interleukin‐18; IL‐18R, interleukin‐18 receptor; IL‐33, interleukin‐33; TNF‐α, tumor necrosis factor alpha; TGFβ, transforming growth factor beta; C1q, complement component 1q; FKN, fractalkine; HMGB1, high‐mobility group box 1; P2XRs, purinergic P2X receptors; P2Yrs, purinergic P2Y receptors; P2×4R, purinergic P2×4 receptor; P2×7R, purinergic P2×7 receptor; P2Y12R, purinergic P2Y12 receptor; P2Y14R, purinergic P2Y14 receptor; TLRs, toll‐like receptors; TREM1, triggering receptor expressed on myeloid cells 1; PAR2, protease‐activated receptor 2; NLRP3, NOD‐like receptor family pyrin domain‐containing 3; CX3CR1, C–X3–C motif chemokine receptor 1; S1PR1, sphingosine‐1‐phosphate receptor 1; CRLR/RAMP1, calcitonin receptor‐like receptor/receptor activity‐modifying protein 1; TRPV1, transient receptor potential vanilloid 1; TRPA1, Transient receptor potential ankyrin 1; Panx1, Pannexin 1; EAAT2, excitatory amino acid transporter 2; Na^+^/K^+^ ATPase, sodium–potassium adenosine triphosphatase;α7nAChR, alpha‐7 nicotinic acetylcholine receptor. Illustrations were created via biorender (http://app.biorender.com).

Taken together, migraine‐associated neuroinflammation is best understood as a distributed neuroglial process whose expression varies by phase, anatomical compartment, sex, and subtype. The strongest evidence currently supports astrocyte–microglia interactions during CSD and central sensitization, together with SGC and Schwann cell participation during trigeminal neuropeptide‐driven peripheral sensitization. However, the field should remain cautious about equating preclinical glial activation with a universal inflammatory state in all patients with migraine. A more precise model is that glial dynamics shape attack susceptibility, amplification, and resolution in a context‐dependent manner, with especially strong relevance in aura and CM (Figure 3).

### 4.5 Phase‐Specific Biological Mechanisms

**FIGURE 4:**
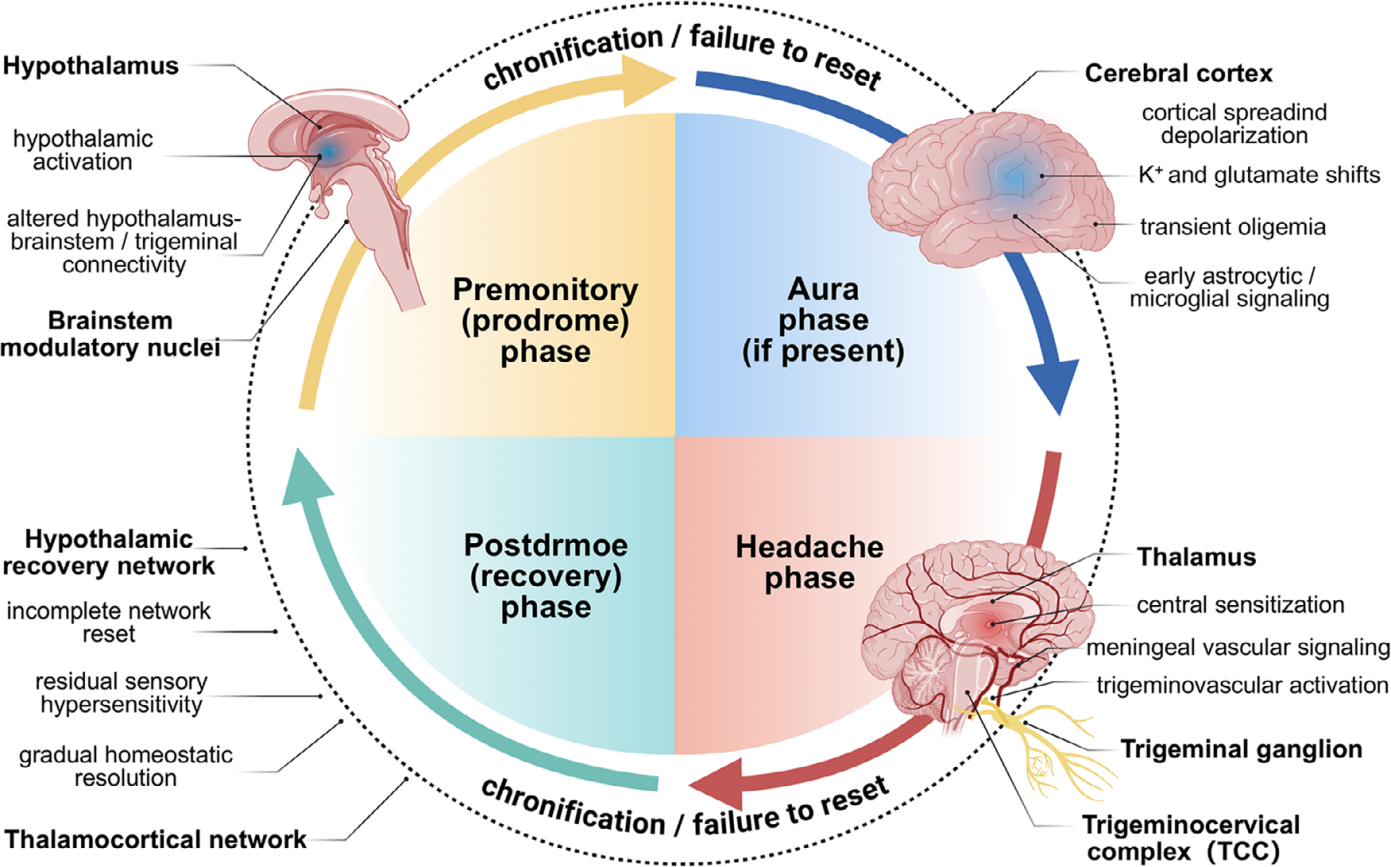
Phase‐specific biological mechanisms of a migraine attack. The figure depicts migraine as a dynamic process unfolding across four partially overlapping phases: premonitory, aura (when present), headache, and postdrome. Early attack biology is centered on hypothalamic and brainstem network dysfunction; in attacks with aura, cortical spreading depolarization provides the best‐supported mechanism for transient neurological symptoms and contributes downstream neuroimmune and trigeminovascular activation. The headache phase is driven by trigeminovascular signaling, including CGRP release, meningeal nociceptor sensitization, and central amplification within the trigeminocervical complex and thalamocortical pathways. The postdrome reflects incomplete recovery of these systems, with persistent fatigue, cognitive slowing, and residual sensory hypersensitivity. Illustrations were created via biorender (http://app.biorender.com).

Migraine is best understood not as a static lesion or a single‐pathway disorder, but as a dynamic biological process that unfolds across partially overlapping clinical phases. Although not every patient experiences every phase in every attack, the premonitory, aura, headache, postdrome, and chronification stages can be mapped onto interacting neural, vascular, and neuroimmune mechanisms. This phase‐based framework helps integrate the distributed brain network model with the trigeminovascular hypothesis and provides a more clinically useful account of how migraine evolves over time [128, 235] (Figure 4).

The premonitory phase likely reflects early dysfunction within hypothalamic, brainstem, and related homeostatic networks before the onset of pain. Symptoms such as yawning, thirst, food craving, fatigue, mood change, and altered arousal suggest disruption of systems governing energy balance, circadian biology, autonomic regulation, and salience processing [128]. Functional imaging studies have shown altered hypothalamic connectivity with brainstem and trigeminal regions in the hours to days preceding headache, supporting the view that attack initiation involves central network changes rather than a purely peripheral nociceptive event [128, 236]. Brainstem nuclei, including the periaqueductal gray, locus coeruleus, rostral ventromedial medulla, and parabrachial complex, are also positioned to shape trigeminovascular gain, autonomic symptoms, and sensory threshold during this phase. Accordingly, some purported “triggers” may in fact represent early manifestations of premonitory network dysfunction rather than external causes of attack initiation [128].

In patients who experience aura, the aura phase is most consistently explained by CSD, a slowly propagating wave of near‐complete neuronal and glial depolarization followed by transient suppression of cortical activity [148]. CSD provides the best mechanistic explanation for the gradual progression and topography of visual and sensory aura symptoms. Beyond its role as the electrophysiological substrate of aura, CSD also induces marked ionic, metabolic, and inflammatory perturbations, including extracellular K^+^ and glutamate shifts, neurotransmitter release, astrocytic and microglial responses, and downstream activation of trigeminovascular pathways in experimental models [134, 148]. The strength of the evidence linking CSD to headache is now greater than older formulations allowed, particularly for MwA, but CSD should still not be presented as a universal mechanism for all migraine attacks because many attacks occur without clinically apparent aura [148].

The headache phase reflects activation and sensitization of the trigeminovascular system, which remains the principal substrate for migraine pain generation. Peripheral trigeminal afferents innervating the meninges and intracranial vasculature converge centrally in the TCC, from which second‐order neurons project to the thalamus, hypothalamus, brainstem, and cortical regions involved in sensory‐discriminative, autonomic, cognitive, and affective aspects of pain [128, 235]. During this phase, release of neuropeptides, particularly CGRP and also PACAP, contributes to nociceptive transmission, vascular and neuroimmune signaling, and amplification of headache‐related symptoms [128, 237]. Peripheral sensitization may enhance meningeal nociceptor responsiveness, while central sensitization within the TCC and thalamocortical circuits helps explain cutaneous allodynia, photophobia, phonophobia, and the spread of pain‐related hypersensitivity [235]. Importantly, although neurogenic inflammation may contribute to attack biology, it should not be treated as a complete explanation of migraine pain, which depends on integrated peripheral input and central sensory network dysfunction [235].

The postdrome is increasingly recognized as a biologically meaningful recovery phase rather than a nonspecific aftermath of pain. Clinically, patients commonly report fatigue, cognitive slowing, mood change, neck discomfort, and residual sensory hypersensitivity after headache resolution. Mechanistically, this phase likely reflects persistence of altered large‐scale network function together with the gradual restoration of homeostatic balance after trigeminovascular activation and, where present, CSD‐related cortical perturbation [148, 235]. Available evidence suggests that the biological processes engaged during the attack do not terminate abruptly when pain improves; rather, thalamocortical, hypothalamic, and brainstem circuits may remain functionally altered during recovery. Experimental data on spreading depolarization also support the idea that transient disturbances in tissue metabolism, glial signaling, and perivascular clearance can outlast the depolarization wave itself, although direct human evidence remains limited and the postdrome should not yet be overinterpreted through any single mechanism [148, 156].

Chronification can be conceptualized as the failure of these phase‐limited mechanisms to fully reset between attacks. Recurrent trigeminovascular activation, repeated recruitment of thalamocortical and brainstem pain‐modulatory circuits, persistent sensory amplification, and maladaptive plasticity may progressively lower the threshold for future attacks and increase the frequency of interictal symptoms [128, 235]. Central sensitization within the TCC and thalamus appears especially important in this transition, and growing preclinical evidence implicates glial cells as active participants in chronification rather than passive bystanders. Microglial activation in the TCC, astrocyte‐related disruption of excitability homeostasis, and neuron‐glia signaling in the TG together support a model in which repeated attacks induce a more persistent neuroimmune and pronociceptive state that sustains allodynia and headache susceptibility [135, 137]. These mechanisms are unlikely to operate identically across all patients, but they help explain why CM is associated with ongoing sensory hypersensitivity even outside the ictal state.

Taken together, these observations support a phase‐dependent model of migraine biology in which hypothalamic‐brainstem dysregulation can precede pain, CSD explains aura when present, trigeminovascular activation and neuropeptide signaling generate the headache phase, postdrome reflects incomplete network recovery, and chronification emerges from cumulative sensitization and maladaptive plasticity. Framing migraine in this way provides a natural bridge to treatment: different interventions can be linked to different stages of the attack cycle, including modulation of early central networks, suppression of CSD susceptibility, blockade of trigeminovascular neuropeptide signaling, interruption of sensitization, and prevention of long‐term chronification.

## 5 Therapeutic Strategies

Therapeutic management of migraine has shifted from empirical symptom suppression toward more individualized and mechanism‐informed care [128, 238]. This section summarizes current and emerging treatment strategies according to their clinical roles and biological rationale. It begins with general principles of acute and preventive treatment, followed by conventional pharmacological options, migraine‐specific therapies targeting the CGRP pathway, gepants, ditans, neuromodulation, and nonpharmacological interventions. The section also discusses treatment considerations in special populations, challenges related to medication overuse, treatment response, adherence, access, and long‐term disease control. By linking treatment approaches to the pathophysiological mechanisms described above, this section aims to clarify how contemporary migraine therapy is increasingly moving toward precision‐based and patient‐centered management.

### 5.1 Principles of Migraine Treatment

Migraine treatment should be individualized and organized around two core goals: effective termination of attacks and reduction of future attack burden. Contemporary management therefore rests on two complementary pillars—acute treatment and preventive treatment—supported by nonpharmacological and educational strategies [238, 239]. Treatment selection should be guided by attack characteristics, monthly headache frequency, disability, comorbidities, prior treatment response, safety considerations, patient preference, and the risk of medication overuse [238, 240]. This approach is preferable to a uniform stepwise model because migraine is clinically heterogeneous and treatment needs vary substantially across patients and over time [239] (Table 2).

**TABLE 2:** Treatment strategies for migraine.

Treatment category	Drug/therapy	Primary mechanism of action	Key efficacy/evidence summary	Main clinical niche/important limitation
Acute treatment	NSAIDs/acetaminophen	Nonspecific analgesia; COX inhibition (for NSAIDs)	Established first‐line option for mild‐to‐moderate attacks; early treatment improves outcomes, and NSAIDs generally have stronger evidence than acetaminophen monotherapy [245, 246].	Best suited to mild‐to‐moderate attacks or combination acute therapy; limited by gastrointestinal, renal, and cardiovascular risk, and lower migraine specificity.
Acute treatment	Triptans	5‐HT1B/1D agonism; inhibition of trigeminovascular neurotransmission and neuropeptide release	Remain the established migraine‐specific standard for moderate‐to‐severe attacks and the most effective acute agents for many patients [239, 244, 245, 247, 248, 249, 251]	Preferred when rapid migraine‐specific relief is needed; limited by vasoconstriction and cardiovascular/cerebrovascular contraindications.
Acute treatment	Gepants (ubrogepant, rimegepant)	CGRP receptor antagonism	Useful nonvasoconstrictive acute alternatives, especially when triptans are ineffective, poorly tolerated, or unsuitable; complementary rather than uniformly superior to triptans [251, 252, 253].	Good option for patients with cardiovascular concerns or prior triptan failure; limited by access, cost, and shorter real‐world experience than older acute agents.
Acute treatment	Lasmiditan	5‐HT1F agonism; central trigeminal modulation without vasoconstriction	Effective migraine‐specific nonvasoconstrictive acute option that supports a nonvascular mechanism for abortive therapy [254, 255].	Useful when triptans are unsuitable; limited by dizziness, somnolence, and postdose driving restrictions.
Preventive treatment	Beta‐blockers	Adrenergic modulation	Established repurposed preventives with continued evidence‐based use, particularly when matched to comorbidity profile [238, 256, 257].	Useful when hypertension, tremor, or anxiety coexist; limited by fatigue, bradycardia, hypotension, and contraindications such as asthma.
Preventive treatment	Topiramate/valproate	Reduced neuronal excitability	Effective repurposed preventives with longstanding efficacy, but tolerability often limits persistence [238, 256, 257].	Appropriate in selected patients, including some with seizure‐related comorbidity; limited by cognitive slowing, weight/metabolic effects, and teratogenicity, especially for valproate.
Preventive treatment	Amitriptyline/venlafaxine	Monoaminergic pain modulation	Useful preventive options when migraine coexists with insomnia, depression, anxiety, or other pain symptoms [238, 256, 257].	Helpful in patients with mood or sleep comorbidity; limited by sedation, anticholinergic burden, blood pressure effects, and adherence issues.
Preventive treatment	Candesartan/flunarizine	Vascular/calcium‐channel related preventive effects	Reasonable preventive alternatives with supportive evidence and continued clinical use as part of individualized oral prevention [238, 256, 257].	Useful when other oral preventives fail or are poorly tolerated; flunarizine availability is region‐dependent and both agents may be limited by adverse effects or contraindications.
Preventive treatment	CGRP‐pathway monoclonal antibodies (erenumab, fremanezumab, galcanezumab, eptinezumab)	CGRP/receptor blockade	Effective in episodic and chronic migraine, including many patients with prior preventive failures; these agents have reshaped modern migraine prevention [2, 258, 259].	Increasingly positioned as early‐line or first‐line migraine‐specific prevention in appropriate patients; limited mainly by access, reimbursement, injection route, and mild class‐specific adverse effects such as injection‐site reactions or constipation.
Preventive treatment	Atogepant	Oral CGRP receptor antagonism	Phase 3 evidence supports efficacy in chronic migraine, with clinically meaningful reductions in monthly migraine days and good short‐term tolerability [2, 260].	Key oral migraine‐specific preventive option for patients who prefer noninjectable therapy; long‐term positioning, sequencing, and discontinuation data are still evolving.
Preventive treatment	OnabotulinumtoxinA	SNAP‐25 cleavage; reduced neuropeptide release and peripheral sensitization	Established preventive therapy for chronic migraine, with cumulative benefit across repeated cycles and efficacy even in patients with medication overuse [9, 238].	Important option for chronic migraine and high headache‐frequency states; limited by office‐based injections, delayed maximal response, and procedure access.
Nonpharmacological treatment	Neuromodulation (sTMS, eTNS, nVNS/tVNS, REN)	Neuromodulation of cortical excitability, CSD susceptibility, trigeminal signaling, and endogenous pain‐control pathways	Evidence supports benefit as adjunctive or alternative therapy, with modest but clinically meaningful effects in selected patients; some devices have acute and/or preventive roles [128, 261, 262, 263].	Useful when medications are not tolerated, contraindicated, or undesirable; limited by device availability, cost, device‐specific evidence differences, and still‐limited long‐term data.
Nonpharmacological treatment	Behavioral therapies (CBT, biofeedback, relaxation training, sleep regulation)	Cognitive‐behavioral and autonomic/self‐regulatory modulation of stress, coping, and sleep‐related attack vulnerability	Best‐supported nondrug interventions for reducing headache burden and disability, especially in patients with anxiety, insomnia, depression, or high psychosocial load [264, 265, 266, 267].	Particularly valuable as part of multidisciplinary care; limitation is the need for trained providers, time, and sustained patient engagement.
Nonpharmacological treatment	Lifestyle strategies (regular sleep, hydration, meal timing, exercise, individualized trigger management)	Stabilization of attack threshold and reduction of behavioral/lifestyle contributors to attack expression	Core foundation of comprehensive care that complements acute and preventive therapy and supports lower headache burden over time [238, 264, 267].	Essential across most patient groups; limitation is heterogeneity of triggers and the need to avoid overgeneralized or overly restrictive advice.
Emerging/investigational therapies	PACAP‐targeted therapy	PACAP ligand/receptor pathway blockade	Most advanced non‐CGRP emerging class; phase 2 proof‐of‐concept evidence supports preventive efficacy for ligand‐targeted therapy, whereas PAC1‐receptor blockade has not succeeded clinically [140, 237, 268].	Investigational only; promising for patients with inadequate response to CGRP‐pathway therapy, but long‐term efficacy, safety, and treatment positioning remain undefined.
Emerging/investigational therapies	PAR2‐directed approaches	PAR2 blockade targeting trigeminal/meningeal neuroimmune signaling and priming	Promising preclinical and early clinical development highlights PAR2 as a potential neuroimmune target, but it is not established care [238, 268, 269].	Investigational only; current role is research‐stage rather than routine clinical practice.

*Abbreviations*: NSAIDs, nonsteroidal anti‐inflammatory drugs; 5‐HT1B, 5‐hydroxytryptamine 1B; 5‐HT1D, 5‐hydroxytryptamine 1D; CGRP, calcitonin gene‐related peptide; mAbs, monoclonal antibodies; CM, chronic migraine; tVNS, trigeminal nerve stimulators; sTMS, single‐pulse transcranial magnetic stimulation; nVNS, noninvasive vagus nerve stimulation; CSD, cortical spreading depolarization; CBT‐I, cognitive‐behavioral therapy for Insomnia.

In practice, treatment success should not be defined only by partial symptom reduction. For acute therapy, the main goals are rapid pain relief, relief of the most bothersome associated symptom, restoration of function, and minimization of recurrence and rescue medication use [239]. For preventive therapy, the goals are reduction in monthly migraine days, decreased disability, reduced reliance on acute medications, and prevention of progression to CM [238, 240]. Headache diaries and regular follow‐up are important for monitoring efficacy, adherence, tolerability, and medication overuse [241, 242].

### 5.2 Acute Treatment

#### 5.2.1 Established Acute Therapies

Acute pharmacotherapy remains the mainstay of treatment for migraine attacks. NSAIDs and simple analgesics are appropriate first‐line options for many patients with mild‐to‐moderate attacks, whereas triptans remain the established migraine‐specific standard for attacks of moderate‐to‐severe intensity or for attacks not adequately controlled with nonspecific agents [243, 244]. NSAIDs such as ibuprofen, aspirin, diclofenac, and naproxen have the strongest evidence among nonspecific agents, whereas acetaminophen may be useful in selected patients but is generally less effective as monotherapy [245, 246]. Early treatment during the headache phase is associated with better outcomes than delayed treatment [239, 246].

Triptans act primarily through 5‐HT1B/1D receptor agonism, inhibiting trigeminovascular neurotransmission and neuropeptide release, while also producing cranial vasoconstriction [247, 248]. They are available in oral, intranasal, and subcutaneous formulations, allowing treatment to be tailored to attack speed, nausea/vomiting, and the need for rapid onset [239, 248]. Subcutaneous sumatriptan is particularly effective when rapid relief is required, whereas nonoral formulations are useful in patients with prominent gastrointestinal symptoms [239, 248]. However, triptans are contraindicated or used cautiously in patients with significant cardiovascular or cerebrovascular disease because of their vasoconstrictive properties [249, 250]. Antiemetics such as metoclopramide or domperidone may be added when nausea and vomiting are prominent [239].

#### 5.2.2 Migraine‐Specific Acute Therapies: Gepants and Ditans

The acute treatment landscape has expanded with the introduction of gepants and ditans, which are particularly valuable when triptans are ineffective, poorly tolerated, or contraindicated. Gepants, including ubrogepant and rimegepant, antagonize the CGRP receptor and provide acute benefit without vasoconstriction [239, 251, 252, 253]. Their place in therapy is best described as complementary rather than universally superior to triptans. Recent comparative analyses suggest that several triptans remain more effective for 2‐h pain freedom in many patients, but gepants offer an important alternative for selected populations, especially those with cardiovascular concerns or prior triptan failure [239, 251].

Lasmiditan, a selective 5‐HT1F agonist, is another migraine‐specific acute therapy that does not cause vasoconstriction [254, 270]. Its efficacy supports the concept that central trigeminal modulation can abort attacks independently of vascular mechanisms. However, dizziness and somnolence are common, and postdose driving restrictions limit routine use in some patients [239, 254, 270]. Thus, lasmiditan is best positioned as an option for patients in whom triptans are unsuitable or ineffective.

#### 5.2.3 Practical Acute‐Treatment Strategy

Acute treatment should be matched to attack severity and patient profile. A stratified approach is often more useful than rigid stepped care, especially in patients with higher disability [239]. Patients should be advised to treat early, use an appropriate route of administration, and avoid frequent use of acute medications, because medication‐overuse headache (MOH) is a major modifiable risk factor for CM [271, 272]. Opioids and barbiturate‐containing agents should not be used routinely because of limited efficacy and high risks of overuse, dependence, and chronification [239]. The emergence of CGRP‐targeted therapies has also begun to blur the traditional boundary between acute and preventive treatment, with early‐phase or prodromal treatment representing a promising but still developing strategy [2].

### 5.3 Preventive Treatment

Preventive therapy should be considered when migraine is frequent, disabling, insufficiently controlled by optimized acute treatment, or when acute medications are used too often [238, 240]. Current recommendations support prevention in patients with four or more monthly headache days, meaningful functional impairment, poor response to acute therapy, or frequent acute medication use; in practice, preventive treatment should be offered to most patients with CM [238, 240]. The goal is not only to reduce attack frequency, but also to improve quality of life, reduce disability, and lower the risk of progression to CM [238, 241].

#### 5.3.1 Traditional Oral Preventives

Traditional oral preventives—including beta‐blockers, topiramate, valproate, amitriptyline, venlafaxine, candesartan, and flunarizine where available—remain widely used [256, 257, 273, 274]. These agents were not originally developed for migraine and are best viewed as repurposed drugs with variable efficacy and often limited tolerability [238, 256, 257]. Their selection should be guided by comorbidities, contraindications, and patient preference. For example, beta‐blockers may be useful in patients with hypertension or anxiety, whereas amitriptyline may be helpful when insomnia or depressive symptoms are prominent [238, 256].

However, these agents are limited by off‐target adverse effects, including fatigue, weight gain, cognitive slowing, mood changes, and poor persistence [256, 257, 273, 274]. It is therefore no longer sufficient to present them as the unquestioned backbone of prevention. They remain useful and often accessible, but contemporary preventive care increasingly includes migraine‐specific options with stronger tolerability and adherence profiles [2, 238].

#### 5.3.2 Migraine‐Specific Preventives: CGRP‐Pathway Monoclonal Antibodies and Gepants

The most important therapeutic advance in migraine prevention has been the development of CGRP‐pathway therapies, the first pharmacological agents specifically designed for migraine [252, 273, 275]. The approved monoclonal antibodies—erenumab, fremanezumab, galcanezumab, and eptinezumab—reduce monthly migraine days and disability in both episodic and CM, including in many patients with prior preventive failures [239, 252, 275, 276]. Their generally favorable tolerability profile, monthly or quarterly dosing, and migraine‐specific mechanism have improved adherence compared with many oral preventives [275, 276].

These agents should not be described simply as late‐line rescue options. Recent reviews increasingly position CGRP‐targeted therapies as early‐line or first‐line options in appropriate patients, although reimbursement systems often continue to impose step‐therapy restrictions [2, 238]. Adverse events are usually mild, with injection‐site reactions and constipation being the most common [239, 276]. Long‐term comparative, withdrawal, and switching data are still evolving, but overall these agents have changed the standard of preventive care [2, 259].

Oral gepants have extended migraine‐specific prevention beyond injectable therapy. Atogepant is now supported by Phase 3 evidence in CM, with clinically meaningful reductions in monthly migraine days and good short‐term tolerability [260]. This is particularly important for patients who prefer oral therapy or wish to avoid injectable agents. Gepants also illustrate a broader conceptual shift in migraine therapeutics: the same mechanistic pathway can now be targeted acutely, preventively, and potentially in prodromal treatment windows [2].

#### 5.3.3 OnabotulinumtoxinA in CM

OnabotulinumtoxinA remains an established, evidence‐based preventive treatment for CM, particularly in patients with high headache frequency, medication overuse, intolerance to oral preventives, or inadequate response to previous prophylactic therapies [9, 238]. It was the first therapy specifically approved for CM, and it retains a distinct role despite the advent of CGRP‐pathway monoclonal antibodies and gepants. Recent reviews continue to position it as a clinically useful option alongside newer migraine‐specific therapies rather than as an outdated predecessor. It is also effective in CM both with and without acute medication overuse [9, 238]. Mechanistically, onabotulinumtoxinA acts as a peripheral neuromodulator rather than simply a muscle relaxant. By cleaving SNAP‐25, it inhibits the exocytotic release of pronociceptive neurotransmitters and neuropeptides from trigeminal afferents, including CGRP, thereby reducing peripheral sensitization and indirectly dampening central sensitization within trigeminovascular pathways [9].

Its efficacy was established in the PREEMPT trials, which also helped define headache days, rather than attack counts, as the preferred endpoint in CM prevention studies [9]. In practice, treatment is administered according to the PREEMPT protocol (155–195 U across 31–39 sites every 12 weeks) [9, 277]. Response is often cumulative; many patients improve only after the second or third treatment cycle, so premature discontinuation should be avoided [9, 238]. Overall, onabotulinumtoxin A remains important because of its proven efficacy, favorable tolerability, and complementary role within modern CM prevention.

#### 5.3.4 Treatment Sequencing, Monitoring, and Discontinuation

Preventive therapy should be started at a low dose when titration is required, assessed after an adequate trial period, and adjusted according to efficacy, tolerability, and patient goals [242]. Headache diaries are useful for quantifying changes in monthly headache days, medication use, and disability [241, 242]. Combination therapy may be appropriate in selected refractory patients, although the evidence base remains less developed than for monotherapy [274, 278].

Discontinuation is less well defined than initiation. In patients who maintain a sustained response for 6–12 months, tapering or discontinuation can be considered, but the decision should be individualized [241]. This issue is particularly relevant for migraine‐specific therapies, for which formal withdrawal and switching data are still limited [2]. Accordingly, treatment duration should be framed as a clinical decision based on disease control, relapse risk, patient preference, and safety rather than as a fixed rule.

### 5.4 Nonpharmacological and Comprehensive Care

#### 5.4.1 Neuromodulation

Noninvasive neuromodulation has become an important adjunct or alternative to drug therapy, especially in patients who cannot tolerate medications, prefer nonpharmacological options, or belong to special populations [261, 262, 279, 280]. Available devices include single‐pulse transcranial magnetic stimulation, external trigeminal nerve stimulation, noninvasive vagus nerve stimulation, and remote electrical neuromodulation [239, 261, 262, 279]. These approaches are generally safe and well tolerated, although their efficacy is often modest and long‐term evidence remains limited [261, 262, 279, 280]. Implantable approaches are more controversial and are generally reserved for highly refractory cases.

A practical advantage of neuromodulation is its minimal systemic adverse‐effect burden, making it attractive when drug interactions, poor tolerability, pregnancy‐related restrictions, or contraindications to vasoconstrictive agents complicate pharmacological management [128, 263]. Mechanistically, these devices are thought to modulate cortical excitability, inhibit CSD, influence trigeminocervical nociceptive traffic, or engage endogenous pain‐control pathways such as conditioned pain modulation [128]. Current evidence and guideline summaries suggest that some devices have roles in both acute and preventive treatment, whereas others, such as remote electrical neuromodulation, are primarily supported for acute use [239, 263]. Overall, neuromodulation should be presented as a useful evidence‐based option within comprehensive migraine care, but not as a universal substitute for pharmacological treatment.

#### 5.4.2 Behavioral and Lifestyle Interventions

Behavioral and lifestyle measures are core components of migraine care rather than optional add‐ons. The best‐supported interventions include CBT, biofeedback, relaxation training, sleep regularization, stress management, and regular physical activity [264, 265, 266, 267, 281, 282, 283]. These approaches are particularly valuable in patients with anxiety, depression, insomnia, pain catastrophizing, or high disability, and they can reduce both attack frequency and headache‐related burden [265, 266, 281, 284].

Lifestyle counseling should focus on regular sleep, hydration, meal timing, exercise, and reduction of clear attack precipitants, while avoiding overstatement of poorly supported dietary claims [264, 267, 282, 283]. The evidence for dietary elimination strategies, acupuncture, and physical therapy is more mixed; they may help selected patients, but should not be presented as universally effective or mechanistically established treatments [265, 285, 286]. Multidisciplinary care involving neurologists, psychologists, physiotherapists, and primary care clinicians is often beneficial, particularly in patients with CM and comorbid symptoms [265, 286].

#### 5.4.3 Education, Diaries, and Prevention of Medication Overuse

Patient education is a core component of effective migraine management and should be incorporated from the first clinical encounter. Patients need a clear explanation that migraine is a chronic neurological disorder requiring both acute and preventive strategies, rather than a condition managed only by repeatedly taking pain medication [239]. Education should therefore cover the distinction between acute and preventive treatment, the importance of early but appropriate use of acute medications, realistic treatment goals, potential adverse effects, and the need to avoid frequent medication use that can precipitate MOH. Headache diaries or calendars are equally important, as they help document attack frequency, severity, associated symptoms, acute medication use, and possible patterns such as menstruation or sleep disturbance, thereby supporting diagnosis, follow‐up, and treatment adjustment [238, 241]. Because MOH is a modifiable risk factor for migraine progression, early recognition, counseling, and, when necessary, withdrawal of overused medications should be treated as central elements of comprehensive care rather than secondary considerations [238].

### 5.5 Management in Special Populations

#### 5.5.1 Children and Adolescents

Migraine in children and adolescents requires individualized management that integrates developmental considerations, school functioning, comorbidity, and family context [287, 288, 289, 290, 291]. Because clinical presentation may differ from that in adults, careful history‐taking and age‐appropriate assessment are essential [287, 288]. Acute therapy often relies on ibuprofen or acetaminophen, while preventive treatment is more limited than in adults and should emphasize behavioral strategies, sleep, hydration, and regular routines [7, 288, 292]. Psychological therapies, especially CBT and biofeedback, are often useful [288, 292]. Parents and schools also play an important role in reducing disability and supporting adherence to treatment plans [7, 288, 292]. Pediatric evidence for newer therapies is expanding, but remains less mature than in adults; therefore, treatment should still prioritize safety, functional recovery, and minimization of unnecessary drug exposure [238].

#### 5.5.2 Pregnancy and Breastfeeding

During pregnancy and lactation, treatment should prioritize maternal function while minimizing fetal or infant risk [293, 294, 295, 296]. Nonpharmacological approaches are first‐line, including sleep regulation, hydration, behavioral strategies, and possibly neuromodulation [293, 296]. Pharmacological treatment requires caution; valproate is contraindicated, and topiramate requires strong caution because of teratogenic risk [65, 297, 298, 299]. Acetaminophen is generally preferred for acute treatment, whereas preventive therapy should be avoided if possible and considered only after careful risk–benefit assessment [65, 293, 294, 295, 297, 298, 299]. Evidence for newer CGRP‐pathway agents in pregnancy remains insufficient, so these should generally be avoided unless compelling circumstances exist [65, 297, 298, 299]. During breastfeeding, treatment decisions should similarly favor agents with the best established safety data and minimal infant exposure [238].

#### 5.5.3 Older Adults

Older adults with migraine present distinctive diagnostic and therapeutic challenges because migraine phenotype often changes with age, while comorbidities, polypharmacy, and age‐related physiological changes increasingly shape management decisions [44]. In this population, migraine may become less stereotypical: attacks are often less intense, more frequently bilateral, and more likely to fulfil criteria for probable rather than definite migraine, although some individuals continue to experience frequent attacks and CM may persist into later life [44, 300, 301, 302]. Because new‐onset headache after age 50 years raises concern for secondary causes, clinicians should maintain a lower threshold for investigation than in younger adults, particularly when presentation is atypical, progressive, or associated with focal neurological symptoms [300, 301, 302]. Treatment should therefore be individualized, with careful attention to cardiovascular disease, renal and hepatic function, cognitive vulnerability, fall risk, and the high likelihood of drug‐drug interactions [239, 303, 304]. This caution is especially important because age‐related pharmacokinetic and pharmacodynamic changes can alter tolerability and exposure to both acute and preventive medications [16, 239, 303, 304].

Nonpharmacological strategies remain important in this age group and may be especially attractive when medication burden is already high; vestibular rehabilitation and ketogenic interventions have shown preliminary promise for vestibular migraine or migraine‐related vestibular symptoms, but the supporting evidence remains limited and should not be overgeneralized [294, 305]. Likewise, newer migraine‐specific therapies, including anti‐CGRP monoclonal antibodies such as fremanezumab, appear promising, but older adults have been underrepresented in clinical trials, so conclusions regarding safety and effectiveness should remain cautious [306, 307]. Ultrasound‐guided stellate ganglion block may represent a potential option in selected refractory cases, but current evidence is still limited and insufficient to support routine use [308]. Careful evaluation of comorbidities such as hypertension, diabetes, vascular disease, depression, sleep disturbance, and other pain disorders is essential, as these conditions can influence both migraine course and treatment selection [15, 309]. Overall, management in older adults should balance efficacy, safety, diagnostic vigilance, and preservation of quality of life.

#### 5.5.4 Menstrual Migraine and CM With Medication Overuse

These two clinical situations warrant explicit discussion because both are common and have immediate therapeutic implications. Menstrual migraine often requires a targeted short‐term strategy, whereas CM with medication overuse requires a structured plan centered on education, withdrawal of overused acute medication, and optimization of preventive therapy [261, 271, 293, 296]. For menstrual migraine, perimenstrual prophylaxis with a long‐acting NSAID or triptan is a reasonable option when attacks are predictable, disabling, and closely linked to the menstrual window [310, 311]. Management should also consider the regularity of the menstrual cycle, the woman's contraceptive needs, and the presence or absence of aura, because these factors can affect both diagnosis and treatment selection [310, 312].

In CM with medication overuse, patient education remains fundamental, as MOH is both a complication of frequent acute treatment and a marker of suboptimal migraine control [313]. Withdrawal of the overused medication remains central to care, but preventive treatment should not be unnecessarily postponed, particularly in patients with substantial disability or a high monthly headache burden [241, 271, 272]. Migraine‐specific preventive therapies, including CGRP‐targeted agents, are especially relevant in this setting because they can reduce monthly headache burden and decrease reliance on acute medications, thereby supporting recovery from medication overuse while improving longer‐term disease control [311]. Overall, both conditions benefit from proactive, phenotype‐specific management rather than a uniform treatment approach.

### 5.6 Emerging Therapeutic Targets

The therapeutic pipeline is expanding beyond CGRP. PACAP‐targeted therapies are the most advanced emerging class and provide proof of principle that migraine can be modified through non‐CGRP neuropeptide pathways [140, 237]. This is important because a substantial minority of patients do not respond adequately to CGRP‐targeted treatments [238]. Mechanistically, PACAP is highly relevant to migraine biology because it is expressed in migraine‐related regions including the hypothalamus, pons, thalamus, trigeminal ganglia, and trigeminal nucleus caudalis, and PACAP infusion can provoke migraine attacks in susceptible individuals [140, 237]. Ligand‐directed approaches now appear more promising than receptor‐selective blockade, since a monoclonal antibody against the PAC1 receptor failed clinically, whereas the anti‐PACAP ligand antibody Lu AG09222 showed Phase 2 efficacy in reducing monthly migraine days [140, 268].

Other targets under investigation include PAR2 and additional modulators of trigeminovascular and neuroimmune signaling [238, 268]. PAR2 is of particular interest because activation on meningeal and trigeminal structures promotes mast cell‐driven inflammatory signaling, allodynia, and priming in preclinical models, and monoclonal antibody approaches are now entering clinical development [268, 269]. More broadly, these candidates illustrate a shift away from empiric repurposing toward mechanism‐based and potentially personalized treatment, although they remain promising rather than established options at present.

## 6 Future Research Directions and Challenges

### 6.1 Exploration of Precision Medicine and Biomarkers

A major priority in migraine research is the transition from a predominantly symptom‐based, trial‐and‐error treatment paradigm to a more precise biologically informed approach. At present, migraine diagnosis still relies chiefly on clinical criteria, which remain essential but do not fully capture the marked heterogeneity of the disorder in phenotype, disease course, and treatment response [98]. Future work should therefore focus on identifying biomarkers that are not only statistically associated with migraine, but also clinically useful for diagnosis, phenotyping, prognostication, and therapeutic stratification. Candidate biomarkers have emerged from several domains, including neuroimaging, provocation studies, genetics, and biofluids such as blood, saliva, and CSF [98, 314]. Among these, CGRP and PACAP are especially important because they are not merely correlates of disease activity but mechanistically relevant signaling molecules that have already informed therapeutic development [98, 238]. However, no single biomarker is currently sufficiently robust, reproducible, and validated for routine clinical use, and future studies must address confounding by age, sex, metabolic state, attack timing, comorbidities, and treatment exposure [314].

Precision medicine in migraine will also depend on better definition of biologically meaningful subtypes. Genetic studies have identified numerous susceptibility loci, but the disorder is largely polygenic and genetically complex, limiting direct translation into bedside decision‐making at present [98]. Beyond genomics, epigenetic, proteomic, and metabolomic approaches may help identify patient clusters with differing mechanisms of central sensitization, trigeminovascular activation, neuroinflammatory signaling, or treatment responsiveness. Multimodal models that integrate clinical phenotype, headache diaries, neuroimaging, patient‐reported outcomes, and molecular data are likely to be more informative than single‐marker approaches [98, 314]. Such efforts should aim not only to predict who will respond to a CGRP‐targeting therapy, but also to identify those at higher risk of progression from episodic to CM, MOH, or persistent interictal burden [238].

The biomarker agenda is closely linked to the search for additional drug targets. Although CGRP‐directed therapies have transformed preventive treatment, a substantial proportion of patients do not respond adequately or cannot access these drugs [238, 268]. For this reason, future work should continue to explore alternative pathways, including PACAP, PAR2, amylin‐related signaling, potassium channel mechanisms, glutamatergic pathways, transient receptor potential channels, and the endocannabinoid system [238, 268]. Early data on PACAP‐targeted therapy are especially encouraging, but confirmation in larger and longer trials is required before its place in clinical practice can be defined [237, 238]. Overall, the future of precision medicine in migraine lies in linking biomarkers to mechanism, and mechanism to treatment selection, rather than merely cataloguing disease‐associated signals

### 6.2 The Vision for Disease‐Modifying Treatments

Another central challenge is whether migraine treatment can move beyond reducing attack frequency toward modifying the long‐term course of disease. Current preventive therapies, including CGRP‐targeting drugs, clearly improve disease control for many patients, but whether they qualify as true disease‐modifying treatments remains uncertain [238]. Disease modification in migraine would ideally imply durable reduction in susceptibility to attacks, prevention of progression from episodic to CM, attenuation of interictal dysfunction, and sustained benefit that persists beyond the treatment period [238]. Achieving this goal will require clearer conceptual definitions and longer‐duration studies than those typically used in registration trials. Traditional endpoints such as a 50% reduction in monthly migraine days remain useful, but future research should also include more ambitious and patient‐centered outcomes, such as sustained low‐frequency disease, reduced disability, lower interictal burden, improved participation in daily life, and decreased risk of chronification [238].

This vision also requires greater attention to the biology of the interictal state. Migraine is increasingly understood not simply as a sequence of isolated attacks, but as a fluctuating brain disorder with abnormalities in sensory processing, network excitability, and pain modulation that extend beyond the ictal period [98]. Better characterization of interictal brain function, including hypothalamic, thalamocortical, and brainstem network dynamics, may help identify the mechanisms by which episodic migraine becomes chronic and may reveal targets for genuinely disease‐modifying intervention [98]. Relatedly, neuroinflammatory and glial mechanisms remain promising but incompletely resolved areas of investigation. These pathways are biologically plausible and supported by preclinical data, yet their precise role in human migraine progression and treatment response requires more rigorous longitudinal study [268, 314, 315]. In this context, multiomics profiling, advanced imaging, and repeated phenotyping over time may help determine whether therapeutic modulation of these systems can alter disease trajectory rather than simply suppress symptoms [314].

Earlier intervention may also prove important. Recent clinical and real‐world evidence suggests that better outcomes may be achieved when effective preventive therapy is introduced before migraine becomes highly frequent or severely disabling [238]. This raises the possibility that earlier use of mechanism‐based therapies in selected high‐risk patients could reduce long‐term disease burden and perhaps limit progression, although this hypothesis still requires direct prospective testing [238]. Therefore, future trials should examine not only efficacy after treatment failure, but also sequencing, timing of initiation, duration of therapy, discontinuation strategies, and rational combinations of pharmacological and nonpharmacological treatments.

### 6.3 Improving Healthcare Accessibility, Implementation, and Patient Education

Scientific advances alone will not meaningfully reduce the global burden of migraine unless access to diagnosis and evidence‐based care improves. Underdiagnosis, undertreatment, and poor access to appropriate preventive therapy remain major international problems [14, 238]. In the CaMEO‐I analysis, fewer than 15% of respondents with migraine in need of medical care traversed the key barriers of consultation, diagnosis, and minimally appropriate treatment, underscoring that the translational gap remains substantial even in high‐income settings [14]. Barriers also exist within primary care, where clinicians play a central role in migraine management but often report insufficient time, competing comorbidities, and variable familiarity with newer treatment options [316]. These findings support the need for implementation research, simplified care pathways, and educational initiatives directed not only at specialists but also at primary‐care clinicians and other frontline providers [14, 316].

Patient education should be considered a core therapeutic intervention rather than a secondary adjunct. Structured education can improve understanding of disease mechanisms, encourage appropriate use of headache diaries, reduce fear‐based avoidance behaviors, and strengthen self‐management of triggers, sleep, stress, and medication overuse [238]. It may also improve adherence and persistence with preventive treatment, which are essential in a chronic fluctuating disorder such as migraine [238]. Future studies should determine which educational models are most effective across different age groups, health systems, and sociocultural contexts, including digital formats, group‐based programs, and integration with patient organizations. At the same time, the increasing use of electronic diaries, remote monitoring, and digital therapeutics offers opportunities for richer longitudinal data collection, but these tools also need rigorous evaluation to ensure that they improve clinically meaningful outcomes rather than simply expanding data capture [317].

Access to newer therapies is an additional challenge. Although international recommendations now attempt to provide both “optimal” and “essential” treatment pathways for settings with differing resources, availability and affordability remain highly uneven across countries [238, 318]. Policy efforts that strengthen reimbursement, expand essential‐medicines strategies, and improve health‐system prioritization of headache disorders are therefore crucial [318]. This is particularly relevant because WHO continues to emphasize the need for coordinated action to improve diagnosis, treatment, care, and health‐system responses for headache disorders globally [318]. Accordingly, future migraine research should not be confined to mechanisms and therapeutics alone; it should also include comparative‐effectiveness studies, health‐services research, implementation science, and cost‐conscious strategies that can make high‐quality migraine care scalable and equitable worldwide.

## 7 Conclusion and Prospects

Migraine is a highly prevalent and disabling brain disorder that imposes a substantial burden not only on affected individuals but also on families, health‐care systems, and society. Over the past decade, important advances in epidemiology, mechanistic understanding, and clinical therapeutics have reshaped the field. Migraine is now more clearly understood as a complex disorder of sensory processing and network dysfunction involving trigeminovascular, brainstem, hypothalamic, thalamocortical, and cortical mechanisms, rather than a simple vascular headache syndrome [1, 2, 128]. This conceptual shift has had direct therapeutic consequences, most notably through the development of mechanism‐based treatments targeting the CGRP pathway, alongside growing roles for gepants, ditans, and evidence‐based nonpharmacological interventions [15, 98, 128].

At the same time, major challenges remain. Migraine continues to be underdiagnosed and undertreated in many settings, preventive therapies remain underused, and access to newer treatments is highly unequal across regions and health systems. Moreover, the heterogeneity of migraine phenotype, comorbidity burden, and treatment response means that many patients still do not achieve satisfactory disease control with currently available strategies. For this reason, future progress will depend not only on expanding therapeutic options, but also on improving biomarker development, refining precision‐medicine approaches, generating stronger long‐term and comparative‐effectiveness data, and strengthening implementation of structured, patient‐centered systems of care.

Overall, the contemporary management of migraine is moving from empirical symptom control toward earlier, more individualized, and increasingly disease‐targeted care. Continued integration of biological insight, rigorous clinical research, and equitable health‐care delivery will be essential if the full global burden of migraine is to be reduced and long‐term outcomes for patients are to be meaningfully improved.

## Author Contributions

W.L., Y.Z., and J.T. drafted and conceived the initial manuscript. W.L., S.L., C.S., and Y.T. drew the figures and arranged the tables. J.T., W.L., and T.P.S. contributed to the design and critically edited the manuscript. Y.S., F.J., and D.J. critically revised and edited the scientific content. All authors have read and approved the final manuscript.

## Funding

This work was supported by grants from the National Key Research and Development Program of China (No. 2024YFC2510103), the National Science Foundation for Distinguished Young Scholars of China (No. 82425019), the Brain Science and Brain‐like Intelligence Technology‐National Science and Technology Major Project (No. 2025ZD0214900), the National Natural Science Foundation of China (Nos. 82271245, 82270503, 82371218, and 82402888), the Science and Technology Bureau of Suzhou (No. SKY2022110), the MOE Key Laboratory of Geriatric Diseases and Immunology (No. JYN202403, KJS2507), the Basic Research Program of Jiangsu (No. BK20255001), the Jiangsu Key Laboratory of Drug Discovery and Translational Research for Brain Diseases, and the Priority Academic Program Development of Jiangsu Higher Education Institutions (No. KJ2022038).

## Ethics Statement

The authors have nothing to report.

## Conflicts of Interest

Author Dongsheng Jiang is an Editorial board member of MedComm. Author Dongsheng Jiang was not involved in the journal's review of or decisions related to this manuscript. The other authors declared no conflicts of interest.

## Data Availability

All used data are within the manuscript.
